# Recent Advances in Organic Dyes for Application in Dye-Sensitized Solar Cells under Indoor Lighting Conditions

**DOI:** 10.3390/ma16237338

**Published:** 2023-11-25

**Authors:** Francesco D’Amico, Bas de Jong, Matteo Bartolini, Daniele Franchi, Alessio Dessì, Lorenzo Zani, Xheila Yzeiri, Emanuela Gatto, Annalisa Santucci, Aldo Di Carlo, Gianna Reginato, Lucio Cinà, Luigi Vesce

**Affiliations:** 1 Institute of Chemistry of Organometallic Compounds (CNR-ICCOM), Via Madonna del Piano 10, 50019 Sesto Fiorentino (FI), Italy; francesco.damico@iccom.cnr.it (F.D.); daniele.franchi@cnr.it (D.F.); a.dessi@iccom.cnr.it (A.D.); lorenzo.zani@iccom.cnr.it (L.Z.); xheila.yzeiri@iccom.cnr.it (X.Y.); gianna.reginato@iccom.cnr.it (G.R.); 2 Department of Biotechnology, Chemistry and Pharmacy University of Siena, Via Aldo Moro 2, 53100 Siena, Italy; bas.de.jong@cicciresearch.it (B.d.J.); annalisa.santucci@unisi.it (A.S.); 3 Cicci Research S.R.L., Via Giordania 227, 58100 Grosseto, Italy; luciocina@cicciresearch.com; 4 Department of Chemical Science and Technologies, University of Rome “Tor Vergata”, Via della Ricerca Scientifica, 00133 Roma, Italy; emanuela.gatto@uniroma2.it; 5 Centre for Hybrid and Organic Solar Energy (C.H.O.S.E.), Department of Electronic Engineering, University of Rome Tor Vergata, Via del Politecnico 1, 00133 Rome, Italy; aldo.dicarlo@uniroma2.it; 6 Istituto di Struttura della Materia (CNR-ISM), via del Fosso del Cavaliere 100, 00133 Rome, Italy

**Keywords:** dye-sensitized solar cells, organic dyes, indoor photovoltaics, co-sensitization, photovoltaic characterization

## Abstract

Among the emerging photovoltaic (PV) technologies, Dye-Sensitized Solar Cells (DSSCs) appear especially interesting in view of their potential for unconventional PV applications. In particular, DSSCs have been proven to provide excellent performances under indoor illumination, opening the way to their use in the field of low-power devices, such as wearable electronics and wireless sensor networks, including those relevant for application to the rapidly growing Internet of Things technology. Considering the low intensity of indoor light sources, efficient light capture constitutes a pivotal factor in optimizing cell efficiency. Consequently, the development of novel dyes exhibiting intense absorption within the visible range and light-harvesting properties well-matched with the emission spectra of the various light sources becomes indispensable. In this review, we will discuss the current state-of-the-art in the design, synthesis, and application of organic dyes as sensitizers for indoor DSSCs, focusing on the most recent results. We will start by examining the various classes of individual dyes reported to date for this application, organized by their structural features, highlighting their strengths and weaknesses. On the basis of this discussion, we will then draft some potential guidelines in an effort to help the design of this kind of sensitizer. Subsequently, we will describe some alternative approaches investigated to improve the light-harvesting properties of the cells, such as the co-sensitization strategy and the use of concerted companion dyes. Finally, the issue of measurement standardization will be introduced, and some considerations regarding the proper characterization methods of indoor PV systems and their differences compared to (simulated) outdoor conditions will be provided.

## 1. Introduction

To tackle the entwined issues of global warming and climate change, one of the mandatory goals that our society will have to reach in the near future is the reduction of CO_2_ emissions [1]. To match this need with the constant increase in global energy demand [2] a radical shift toward extensive energy production from renewable sources will be necessary. In this context, the exploitation of solar energy appears very attractive since it is abundant, widely available, free, and essentially inexhaustible [3].

Harvesting and conversion of solar light into electricity can be realized by photovoltaic (PV) technologies, whose global market is presently dominated by crystalline silicon modules [4]. The latter have been the subject of continuous and innovative research in recent years, leading to the development of ever more efficient and stable devices and to a sustained growth of the global installed PV capacity [5]. Nevertheless, there is still a tremendous need to further extend the diffusion of PV technologies; to reach such a goal, it will be necessary to focus not only on increasing the devices’ power conversion efficiencies (PCE) and extending their lifetime, but also on expanding their use to new applications. In this regard, there are some drawbacks associated with classical Si-based PV systems, particularly due to their poorly appealing aspect, their rigidity and weight, and their limited photovoltaic performances in low light intensities. To overcome these issues, significant research efforts have been devoted to the development of new and emerging PV technologies, including organic (OPV), dye-sensitized (DSSCs), and Perovskite (PSCs) solar cells [6].

**Figure 1:**
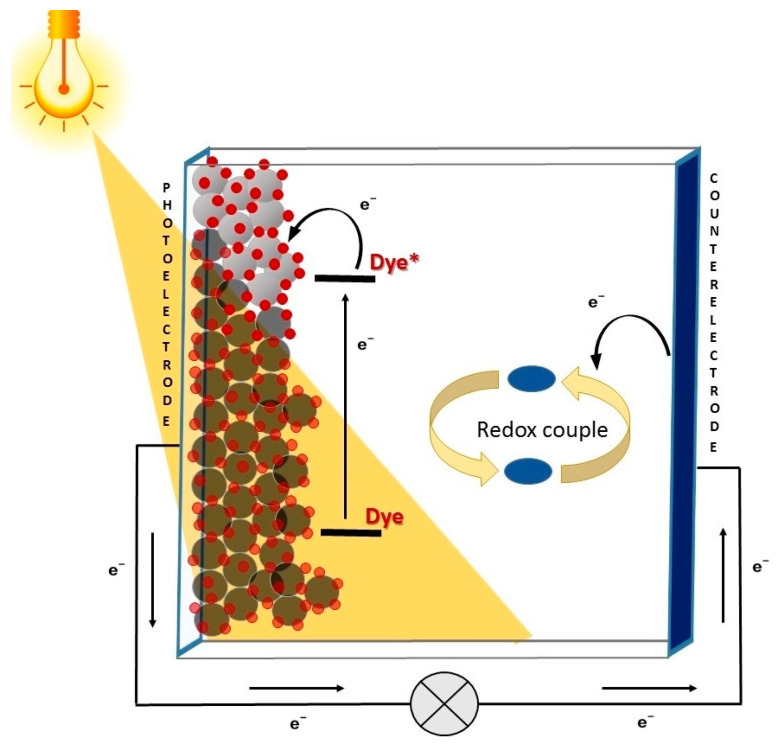
Working mechanism of a DSSC.

Among them, DSSCs are considered particularly suitable for employment in non-traditional settings, thanks to their composition and the peculiar mechanism of electricity generation [7]. The working principle of a DSSC is inspired by natural photosynthesis and is based on the use of a molecular sensitizer, which is responsible for the light harvesting and charge separation processes. Such dye is usually adsorbed on a thin layer of a large band-gap semiconductor (such as TiO_2_) coated on a conductive substrate, constituting the cell photoanode. When a photon is absorbed by the sensitizer, an electron is excited from its highest occupied molecular orbital (HOMO) to its lowest unoccupied one (LUMO). If the energy levels are correctly aligned, the electron is then transferred (“injected”) into the conduction band of the semiconductor layer, and from there, it reaches the conductive substrate. After charge collection at the electrode, the electrons then travel to the counter-electrode through an external circuit, thus producing an electric current. The regeneration of the dye is finally carried out by a suitable redox couple, whose role is to shuttle charges between the two electrodes (Figure 1) [8].

Thus, the basic components of DSSCs simply consist of a photoanode made of glass coated with a transparent conducting oxide film, a dye, an electrolyte containing a redox couple, and a counter electrode. The latter is usually made of a thin film of metallic platinum, but recently some more available and cost-effective alternatives have been proposed [9,10,11]. As a consequence, DSSCs are characterized by the facility of fabrication, abundance and low cost of the materials employed, and low environmental impact [12,13]. Thanks to their structure, they present various advantageous features [7], such as tunable color, low weight, and good transparency, and their performances are scarcely dependent on the angle of incident light (i.e., they work well also under diffuse light).

For the above reasons, DSSCs can, for instance, be easily integrated into buildings to replace roof tiles or to cover building façades [14,15]. In addition, they can also be used in agrivoltaics [16,17,18], for instance by installing them on greenhouse roofing. Indeed, it has been shown that cells built using dyes that absorb light only in the green region of the spectrum, which is the less involved in the most common photosynthetic processes [19], can produce electricity without interfering with plant growth [20,21]. Finally, DSSCs have emerged as one of the best PV technologies for power generation under indoor light conditions, where they have shown their highest potential, reaching PCEs of up to 34% [22]. Such figures are clearly higher than the PCEs usually reported for (simulated) outdoor conditions and are enabled on one hand by the capability of dye-sensitized photoanodes to efficiently harvest diffused light, and on the other by the different spectral distribution of common indoor light sources compared to the Sun, for which it has been demonstrated that a higher theoretical efficiency can be attained for lager bandgap values (1.8–2.0 eV), comparable with those of typical DSSC dyes [23]. Nevertheless, it is important to keep in mind that, despite the excellent PCE values, the power generated by DSSCs in dim light conditions is always very low, as a consequence of the low intensity of the incoming radiation (see below) [24].

The above-mentioned outstanding properties contributed to revamping the interest in DSSC, foreshadowing the possibility of a wide range of alternative applications such as the powering of wearable electronics, wireless sensor networks, and low-power devices. In this context, it has been estimated that under indoor light conditions, DSSCs should be able to produce a power intensity of >100 μW cm^−2^, which is adequate to power electronic devices with applications in the so-called “Internet of Things” (IoT) [23]. IoT is a recently-introduced scenario describing a network of physical objects, i.e., “things”, embedded with sensors, software, and wireless communication modules, capable of connecting and exchanging data with other devices and appliances over the internet [25,26,27]. The number of studies examining the various aspects of IoT has grown rapidly in recent years, and in the near future, such a concept is expected to find its implementation in many sectors, as for instance human health, smart homes and cities, standardized production environments and smart offices, leading to significant benefits in terms of comfort of the indoor environment, resources optimization, and energy savings [28,29]. As a result, billions of IoT devices are expected to be located inside buildings and will need to be powered by batteries or grid connections. Clearly, the use of indoor PV, and DSSCs in particular, as alternative renewable power sources for IoT devices might contribute to make future wireless networks and sensors autonomous and grid-independent, improving their reliability and sustainability. Consequently, the discovery of new stable, low-cost, efficient, and sustainable materials, capable of boosting DSSC performances under dim light, will be essential to satisfy the expected growth in demand for IoT applications [30,31], possibly in combination with other technologies, such as those based on perovskite PV [32,33].

From the above discussion, it emerges clearly that DSSCs, thanks to their host of different applications, can play a significant role in the decarbonization of the energy sector [34], for example by reducing the need to produce, replace, and possibly recycle unsustainable batteries [35].

Among the components of DSSCs, the sensitizer obviously plays the most important role in light harvesting [36,37]. Taking into account that most indoor light sources are constituted by fluorescent lamps or LEDs whose emissions are localized at wavelengths ranging from 400 nm to 700 nm and that the intensity of incident indoor light is typically three orders of magnitude less than that of sunlight [38], accurate engineering of dyes, ensuring a good spectral match with the chosen light source and a high molar attenuation coefficient, becomes crucial to maximizing device efficiency.

From this point of view, organic dyes appear very attractive thanks to their relatively facile preparation and purification procedures, coupled with easily tunable spectro-electrochemical properties [36]. In particular, most organic dyes used in DSSCs are based on a molecular donor–π bridge–acceptor (D–π–A) design, which has been shown to yield good charge separation properties, high molar extinction coefficients in the visible region and good photo- and chemical stability [39]. Indeed, some reviews have already appeared in the literature in 2020 and 2021 concerning the indoor application of this kind of sensitizers [40,41,42,43].

Currently, this research field is evolving at a very fast pace and, consequently, several further advances have been reported in the last few years, which will be presented in this review. In particular, we will discuss the current state-of-the-art in the design and application of organic dyes for indoor PV, some of which have been found very promising. Dyes will be classified with respect to their central scaffold. Two specific chapters will be also dedicated to the discussion of the co-sensitization approach and the new family of concerted companion dyes (CCD), respectively.

In addition, a key issue in the real exploitation at the industrial and commercial levels of the DSSC technology is the upscaling from small lab-scale cells to large area modules and panels by sustainable fabrication processes [44,45,46]. Since the sheet resistance of the TCO (transparent conductive oxide) substrate causes ohmic losses, the module is composed of a number of narrow cells connected in series or parallel thanks to a conductive grid [47,48]. Accordingly, throughout the text, we will present some examples of upscaling of DSSC modules for indoor applications according to the different adopted dyes.

Finally, it must be highlighted that comparing the indoor performances of DSSC measured in different studies is still very challenging, due to the enduring lack of universally accepted standards for indoor spectral quality and integrated irradiance. In addition, the way in which several variables that influence IPV measurements [49], such as system design, spectral response nature of the active material, and temperature, are controlled can vary from study to study. Clearly, establishing a universal protocol for measuring and comparing indoor DSSC performances represents a mandatory milestone toward the large-scale deployment of this technology, contributing to meet the expected future market request. To address this issue, some considerations regarding the proper characterization methods of indoor PV systems and their differences compared to (simulated) outdoor conditions will be provided at the end of the manuscript.

The results summarized in this review highlight the extreme vitality of research in the area of dye-sensitized solar cells for indoor and low-light applications, which is currently witnessing some impressive developments. In addition, this manuscript combines a fundamental discussion of the dyes’ molecular properties and their impact on device characteristics with more practically oriented considerations, regarding the scale-up of PV modules and the correct procedures for their characterization. As such, we hope that it will constitute a useful tool for all researchers involved in this exciting field.

## 2. Recent Developments in the Design and Synthesis of Organic Sensitizers for Indoor DSSC Applications

In the first section of this review, we will discuss recent reports concerning the application of new organic dyes used individually as sensitizers for indoor DSSCs. Despite the growing number of studies published in the last few years, it must be pointed out that research in this area was mostly carried out by a few research groups focusing only on the development of specific compound classes, and that a systematic comparison of performances across a broad range of structurally diverse compounds is still lacking. As a result, it is currently difficult to identify general development trends or the emergence of “privileged” structures, if not within the body of work published by a single research group.

For this reason, compounds will be simply classified based on their central conjugated units, namely (1) fluorene-based dyes, (2) anthracene-based dyes, (3) benzopyrazine- and thienopyrazine-based dyes, followed by a section describing miscellaneous dyes not belonging to those categories. Finally, a short conclusive paragraph will be presented, in which we will provide some potential guidelines for the future design of this kind of sensitizers.

### 2.1. Fluorene-Based Dyes

**Figure 2:**
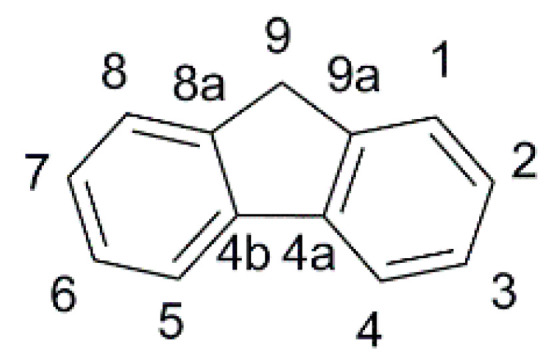
The fluorene scaffold with the numbering of the ring positions.

In the field of indoor DSSC, the fluorene scaffold (Figure 2) and its derivatives were explored by Chow, Chang, and co-workers. They reported several dyes where such motif was used as an internal π-spacer [50] or derivatized into internal acceptor moieties [51], to obtain a library of fluorene-based dyes, featuring aromatic and aliphatic tertiary amines or phenothiazine donor groups, characterized by a fine-tuning of the frontier orbitals’ energies. Such systematic study of the interaction of fluorene with phenyl or thiophene rings directly connected as π spacers supported the rationale for the effect of torsion angles on the optical and electrochemical properties of this class of dyes.

**Figure 3:**
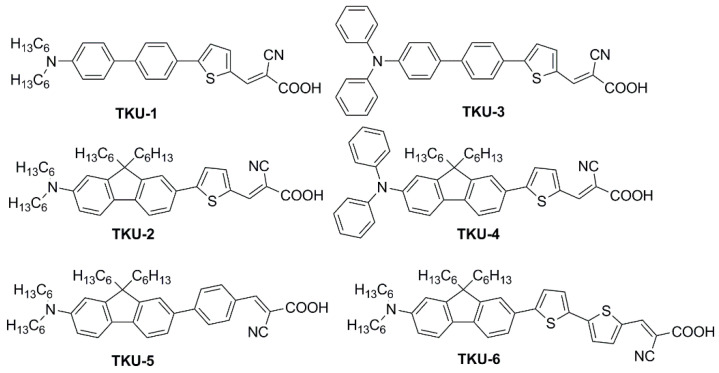
Dyes containing a fluorene moiety as a replacement for a biphenyl spacer [52].

In the first report of indoor application of fluorene-containing dyes [50], a series of D-π-A structures was described, where the fluorene moiety was introduced to replace a biphenyl spacer (present in model dyes **TKU-1** and **TKU-3**, Figure 3) aiming to increase the rigidity of the conjugated bridge.

**Figure 4:**
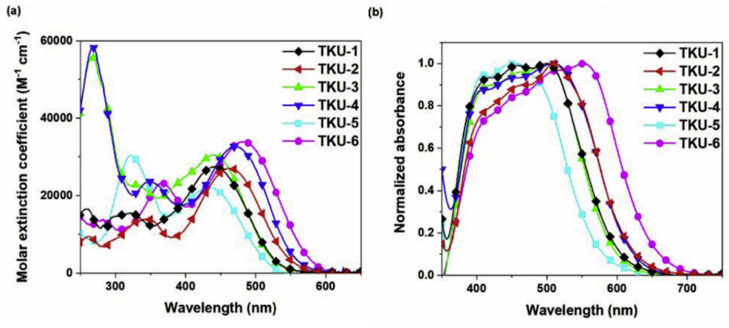
UV–Vis spectra of dyes **TKU-1**–**TKU-6** (**a**) in THF solution (ca. 10^−5^ M) and (**b**) on TiO_2_ surface. Reproduced from. Ref. [50] with permission from Elsevier.

The UV-Vis absorption spectra of the dyes in THF solution are shown in Figure 4a. All compounds presented a localized π-π^∗^ transition band below 400 nm, accompanied by an intramolecular charge transfer (ICT) transition band located in the visible region between 430 and 500 nm. Interestingly, the absorption features were influenced by the nature of the π-bridge section. Thus, varying the π-scaffold from biphenyl (**TKU-1** and **TKU-3**) to fluorene (**TKU-2** and **TKU-4**), the absorption spectra in solution were red-shifted thanks to a superior light-harvesting ability, in turn due to a more planar structure. The same effect was observed going from a structure where fluorene was directly connected to a phenyl ring (**TKU-5**) to one where it was linked to thiophene (**TKU-2**), and even more to a bithiophene moiety (**TKU-6**), thanks to a reduction of the dihedral angle between the aromatic planes. On the other hand, UV-Vis absorption spectra of the dyes adsorbed on TiO_2_ were measured in the hope of demonstrating the anti-aggregating effect of the hexyl chains placed on the tridimensional sp^3^ C9 site of the fluorene moiety. However, this proved not to be the case, as the absorption bands were strongly red-shifted and broadened compared to those of the samples in the THF solution, meaning that the alkyl chains were not bulky enough to prevent aggregation (Figure 4b).

DSSCs built with biphenyl dyes (**TKU-1** and **TKU-3**) were found to have longer lifetimes of the injected electrons than those fabricated with the fluorene-based analogs (**TKU-2** and **TKU-4**). The same was observed for π-spacers directly connected to a phenyl ring (**TKU-5**) instead of thiophene (**TKU-2**), correlating this behavior to their more twisted molecular structure. The authors suggest that such twisted moieties can form a blocking layer that shields the TiO_2_ surface from the electrolyte, therefore reducing electron recombination. The above assumptions were found in agreement with the photovoltaic properties of the devices tested under AM 1.5 G irradiation conditions: dyes with planar moieties produce larger *J_SC_* (**TKU-2** > **TKU-1**, **TKU-4** > **TKU-3**, **TKU-6** > **TKU-2** > **TKU-5**) while twisted moieties produced higher *V_OC_* (reverse order).

All six **TKU** dyes were also exploited for the fabrication of indoor devices using an electrolyte with a low content of iodine so as not to hamper the light absorption of the dye. When comparing the six dyes, a different trend of the *V_OC_* was observed depending on the illumination conditions: low-intensity illumination (600 lux LED, or 600 lux T5 lamp) or simulated sunlight (AM 1.5 G condition) (Table 1).

Interestingly, the authors were able to correlate it with the different bulkiness of the dyes: under low light intensity, **TKU-1** and **TKU-3**, featuring only a few bulky substituents, display the largest drop in *V_OC_* with respect to the simulated sunlight irradiation. This suggests that charge recombination phenomena play a significant role for the DSSCs under low lighting intensity, more than under simulated sunlight conditions. This observation led the author to conclude that the best-performing dyes under (simulated) sunlight are not necessarily also the ideal ones under indoor light conditions and that their behavior should not be related only to the spectral overlap between the light source emission and the dye absorption, but also to the efficacy in shielding the TiO_2_ surface from the electrolyte.

**Figure 5:**
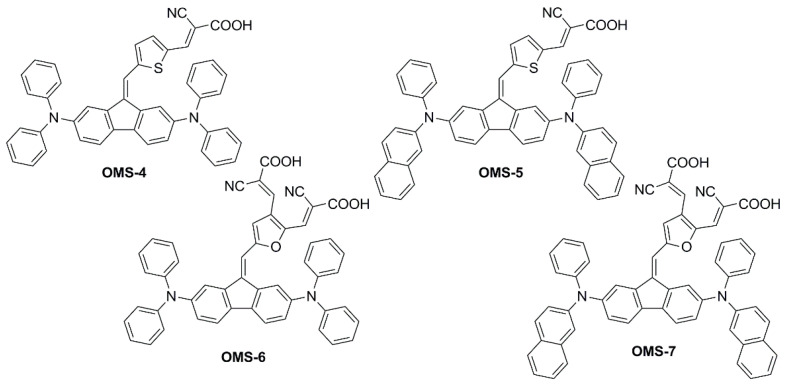
Structures of multi-branched dibenzofulvene-based organic dyes [51].

In a subsequent report [51], the same authors explored the design of four multi-branched dyes featuring a dibenzofulvene unit connected with thienyl or furanyl as a conjugation bridge, T-shaped diarylamines (diphenylamine or phenyl-1-naphthylamine) as donors and different numbers of cyanoacrylic acid moieties as anchors (**OMS-4**–**7**, Figure 5).

**Figure 6:**
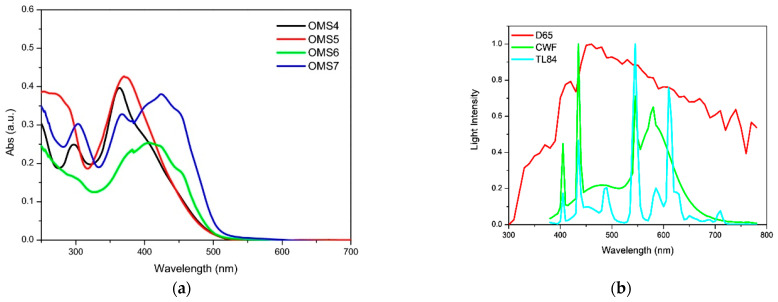
Absorption spectra of the **OMS** series dyes in THF solution (**a**) and emission spectra of different indoor light sources (**b**). Reproduced from Ref. [51] with permission from Springer.

This was exploited to allow an electronic connection between the donor moieties located on the aromatic core and the acceptor/anchoring function on the sp^2^-hybridized C9 position. Indeed, all compounds exhibited UV-Vis absorption spectra with a significant component in the visible region (Figure 6), which was red-shifted for the dyes having a double acceptor group and a phenyl-1-naphthylamine donor. The authors report that emissions were very weak for the entire series of dyes, and thus band gaps were estimated from the onset of the absorption spectra.

Other than improving optical properties, the dye structure was expected to be bulky enough to efficiently suppress intermolecular interactions and charge recombination phenomena in the cells. However, the lack of electron lifetime measurements and device efficiency measurements in the absence of the disaggregating additive chenodeoxycholic acid (CDCA) did not allow for proving this assumption. On the other hand, it was shown that the excessively planar structure was detrimental to device *V_OC_*. Compared to thiophene, when furan was directly connected with dibenzofulvene (dihedral angle with furan: 9°; with thiophene: 30°) high rates of recombination were observed for the corresponding cells under simulated sunlight, causing the *V_OC_* of the devices to drop (*V_OC_* = 0.63 and 0.55 V for **OMS-4** and **OMS-6**, respectively; *V_OC_* = 0.61 and 0.55 V for **OMS-5** and **OMS-7**, respectively).

Unfortunately, in this work, only the best-performing dye under AM 1.5 G simulated sunlight (**OMS-4**) was tested under indoor lighting, preventing a comparison of the efficiency trends under different conditions, as performed in the previous study [50]. Despite the dye absorption spectrum having a larger overlap with the emission of a simulated diffuse illumination standard (D65) than with that of a fluorescent lamp (CWF) (Figure 6), **OMS-4**-based devices showed the highest efficiency under the latter (PCE = 2.48% and 7.26% under 1000 lux D65 and 1000 lux CWF, respectively), highlighting once again that the efficiency of indoor devices is the result of several competing factors and is not only related to the spectral overlap between the light source emission and the dye absorption.

Finally, even though a DFT analysis highlighted the expected ICT upon photoexcitation, the authors noticed that the *J_SC_* of all devices was relatively low and addressed such behavior to the unfavorable 2,7-positions of the donor moiety in the benzofulvene system, preventing proper conjugation with the rest of the unsaturated scaffold of the dyes.

**Figure 7:**
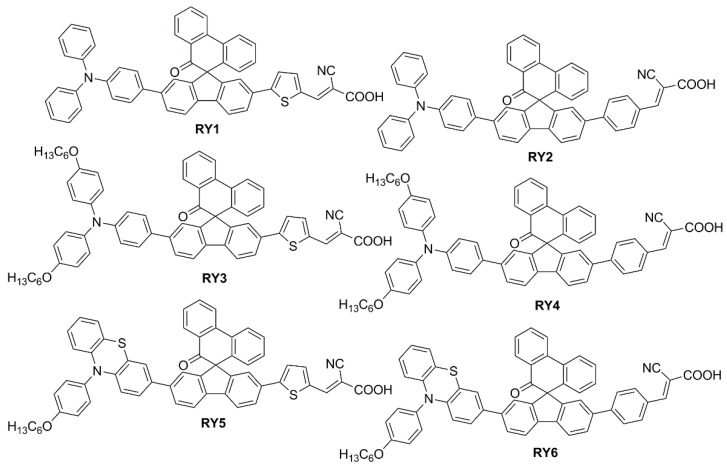
Structures of spiro-phenanthrenone-based organic dyes **RY1-6** [53].

To modify the electronic and steric properties of the π spacer, the same research group reported also a small collection of dyes (Figure 7) containing a spiro-phenanthrenone core [52], which was supposed to act as an auxiliary acceptor moiety that, at the same time, could efficiently suppress intermolecular aggregation and prevent the electrolyte from reaching the TiO_2_ surface, thanks to its bulky three-dimensional structure.

**Figure 8:**
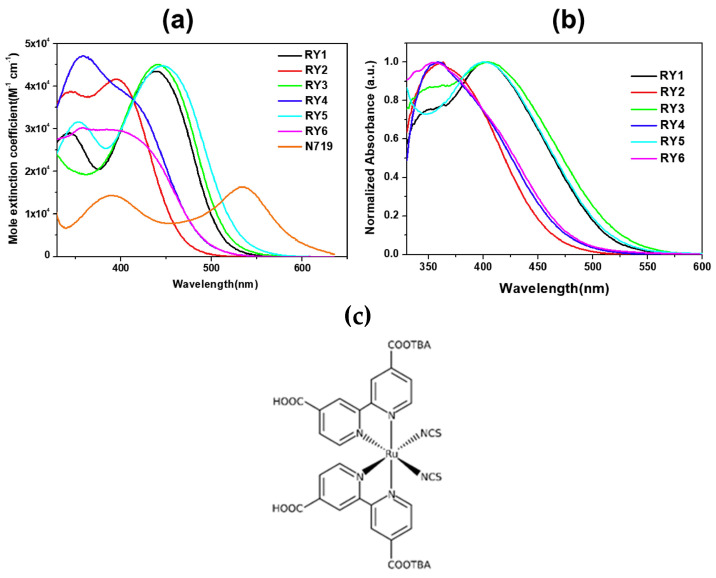
(**a**) Absorption spectra of dyes **RY1**–**RY6** and **N719** in a MeCN/*t*BuOH 1:1 mix (3.0 × 10^−4^ M); (**b**) Absorption spectra of dyes **RY1**–**RY6** adsorbed on TiO_2_. Reproduced from Ref. [53] with permission from Elsevier. (**c**) Structure of metal-organic dye **N719**.

The UV-Vis absorption spectra of the dyes in a MeCN/*t*BuOH 1:1 solution are reported in Figure 8a, demonstrating the red-shifting effect of the thiophene unit (**RY1**,**3**,**5**) in comparison to the phenyl ring (**RY2**,**4**,**6**) connected to the anchor, with the maxima of the ICT bands located around 450 nm. When adsorbed on TiO_2_, the ICT transitions exhibited a slight blue-shift compared to those in solutions, which was ascribed to the deprotonation of the cyanoacrylic acid, but the difference between the two series of dyes remained clear.

As expected from the above design considerations, DSSCs built with such spiro-fluorene dyes and tested under AM 1.5 G conditions were found to lose efficiency when CDCA was used in the staining procedure, which was mainly due to a drop in photocurrents not compensated by the little gain in *V_OC_*. This was interpreted as a piece of evidence for the spiro-fluorene dyes to be able to promote vertical alignment and good dye coverage on TiO_2_ by themselves. Then, DSSCs were prepared with all the dyes of the series and tested under TL84 European fluorescent light and, in some cases, CWF cool white light as well. The different efficiencies recorded (see Table 2) were correlated to the different overlaps of the absorption spectra of the dyes and the emission profiles of the lamps.

Stability tests were also conducted on cells sensitized with dye **RY3**, either alone or co-sensitized with Ru-based dye **N719** (Figure 8c). Both devices proved sufficiently stable, retaining 90% and 93% of their initial efficiency over 192 h indoor aging test, respectively.

**Figure 9:**
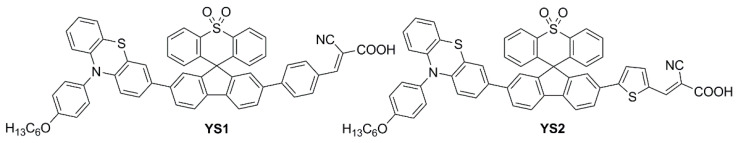
Structures of thioxanthenedioxide spiro fluorene-based organic dyes **YS1**,**2** [53].

In a more recent paper, the same group also explored new electron-poor polycyclic moieties to functionalize the fluorene at the C9 site [53]. Thioxanthenedioxide spiro fluorene was used as the auxiliary acceptor moiety in two dyes (**YS1** and **YS2**, Figure 9), in the hope of exploiting the interaction of the sulfone group with the Li^+^ ions present in the liquid electrolyte to trap them and block the surface of TiO2. This was expected to increase the Fermi level of the semiconductor and thus enhance cell *V_OC_* [54]. At the same time, the bulkiness of the central unit was anticipated to trap I_3_^−^ ions and retard charge recombination.

The weakening of the S=O double bond due to the interaction with Li^+^ ions was observed by the disappearance of the corresponding stretching peak in the FT-IR spectra. In indoor DSSCs, the new dyes were only used as co-sensitizers of **N719**, reaching PCE values of 20.82 ± 0.49% and 23.76 ± 0.96% for **YS1** and **YS2**, respectively, under 2500 lux TL84 fluorescent light.

In summary, the above studies indicate that fluorene is a planar spacer with large conjugation that can foster broadband absorptions and allow good electron connections between donor and acceptor moieties in DSSC sensitizers, but only if the latter are located in positions 2 and 7 of the fluorene ring system, respectively. Position 9 is instead appropriate for steric and electronic modifications, especially spiro/derivatization with bulky polycyclic substituents. On the other hand, the ICT is not so effective between position 9 and positions 2 and 7. A common finding is that a thienyl π-spacer directly linked to fluorene can further broaden the absorption spectrum, while phenyl rings are less effective. The lower dihedral angle between the planes of fluorene and the adjacent thiophene reduces the HOMO-LUMO energy gap, resulting in red-shifted light absorptions and larger photocurrents. However, the thiophene insertion is usually accompanied by a lower photovoltage that can be ascribed to a detrimental interaction of sulfur atoms with iodine-based electrolytes and faster recombination rates.

### 2.2. Anthracene-Based Dyes

**Figure 10:**
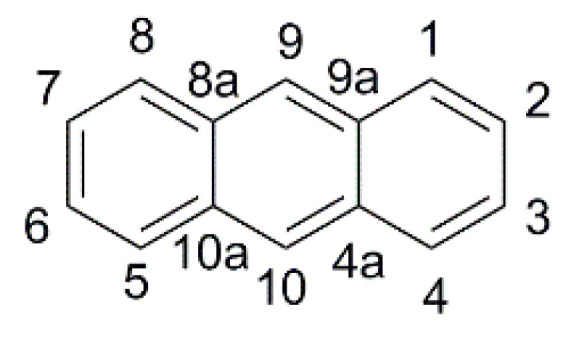
Anthracene scaffold with numbering of the ring positions.

The thermal stability of the anthracene core and the ease of derivatization in 9- and 10- positions (Figure 10) led to its application as a π-spacer in photosensitizers for DSSCs since 2009 [55], but only in 2015 the properties of anthracene-based dyes have been investigated under dim-light irradiation.

**Figure 11:**
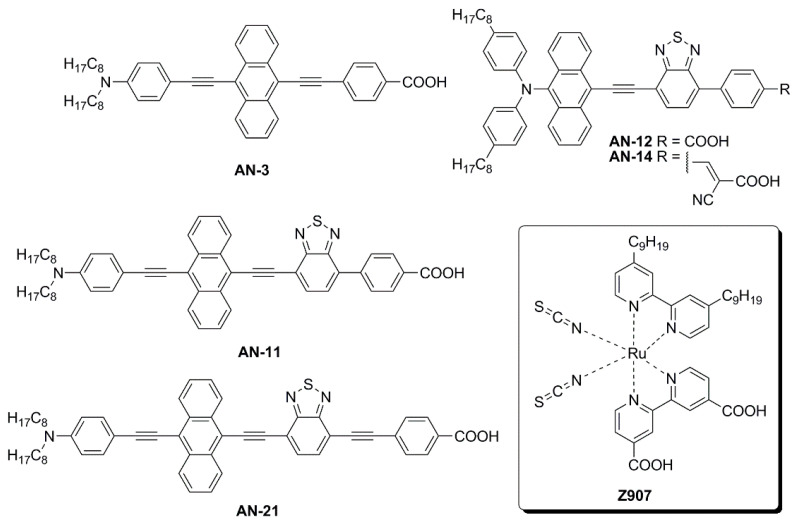
Structures of anthracene-based dyes used under indoor lighting and of dye **Z907** [55,57,58].

The first study in this field was carried out by Lin and co-workers, who reported the synthesis and characterization of five new anthracene-based dyes [56]. After a first screening of the photovoltaic properties of the corresponding DSSCs under simulated AM 1.5 G irradiation (100 mW/cm^2^), the **AN-3** dye (Figure 11) was selected as the most promising one to be investigated under indoor conditions. **AN-3** was synthesized in a very straightforward way through a series of consecutive Sonogashira cross-coupling reactions, resulting in an estimated gram-scale production cost of around 65 USD/g, and exhibited a quite intense light absorption spectrum, mostly centered in the visible region, between 400 and 550 nm. **AN-3**-containing DSSC modules (15 cells each) with an active area of 36 cm^2^ were fabricated using an iodide-based gel as the electrolyte and the corresponding photovoltaic properties were investigated under irradiation by three different artificial light sources (T5, T8, and LED) at 200, 600, and 1000 lux, and compared with analogous DSSC modules containing an organometallic dye (**Z907**—Figure 11) [57] as the reference. The two dyes showed comparable photovoltaic performances under indoor conditions at every illuminance (PCE = 2–3% at 200 lux; 3–5% at 600 lux, 4–6% at 1000 lux): interestingly, under T5 fluorescent light at 200 and 600 lux, **AN-3** even surpassed the efficiency of **Z907**, probably because of the good overlapping between the light absorption spectrum of the dye and the T5 emission spectrum, which in this case proved to be a decisive factor.

**Figure 12:**
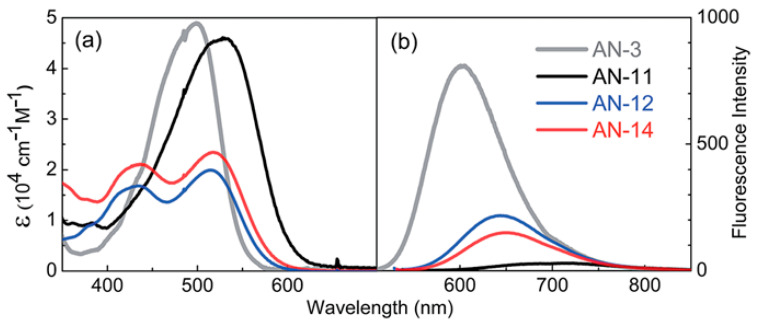
(**a**) Absorption and (**b**) fluorescence emission spectra (2.0 × 10^−6^ M) of the AN dyes in THF solution. Reproduced from Ref. [58] with permission from the Royal Society of Chemistry.

Later, Lin’s group modified the structure of **AN-3** to enhance the push-pull properties of the dye, introducing an additional benzothiadiazole (BTDA) group as an electron-withdrawing motif (**AN-11**—Figure 11) and/or an *N*,*N*-diarylamino-anthryl group as the donor, with a carboxylic (**AN-12**) or a cyanoacrylic acid (**AN-14**) as the anchor [58]. The combined effect of both the elongation of the π-structure and the introduction of an additional acceptor group in **AN-11** was evident in the comparison of the light absorption spectra of **AN-3** and **AN-11** (Figure 12a), since the dyes showed similar molar attenuation coefficients (ε) and comparable absorption bands, with a red-shift of around 30 nm for **AN-11** compared to **AN-3**. On the other hand, the simultaneous modification of both the electron-donating and electron-withdrawing groups in **AN-12** and **AN-14** led to completely different absorption spectra for these two dyes, showing two well-resolved, but less intense absorption bands. The emission bands (Figure 12b) follow the same wavelength trend observed for the lowest energy absorption peaks, that is **AN-11** > **AN-14** > **AN-12** > **AN-3**, but with different intensities, which could be related to different degrees of molecular aggregation of the dyes. Among this dye series, **AN-11** emerged as the sensitizer with the best light-harvesting properties (the most red-shifted *λ_max_*, the highest *ε*, the broadest light absorption spectrum—Figure 12), both in solution and after adsorption on TiO_2_, which affected the photovoltaic performance of the corresponding small-scale DSSCs (active area = 0.25 cm^2^) under both T5 and LED fluorescence light at 1000 lux. Accordingly, **AN-11** gave rise to PCEs of approximately 10%, while **AN-12** and **AN-14** showed poorer efficiencies around 5–6%.

For this reason, a scale-up from small DSSCs to rigid (active area = 26.80 cm^2^) and flexible modules (active area = 19.80 cm^2^) containing the **AN-11** dye was realized: both under T5 and LED illumination, the rigid **AN-11**-containing DSSC modules exhibited PCE values among 9 and 12%, always surpassing the performance of the corresponding **Z907**-containing modules, especially thanks to higher photocurrents, which in turn can be justified by the much greater molar attenuation coefficient and the broader light absorption spectrum of **AN-11** compared to **Z907**. The best PCE value (11.94%) was recorded by **AN-11** under T5 illumination at 1000 lux. Similar behavior was proven also for the flexible modules, even if the average PCE values were a bit lower, between 8–10%. Finally, both rigid and flexible **AN-11**-containing modules were proven to retain 87–90% of their initial PCE values after 600 h of T5 irradiation at 200 lux.

**Figure 13:**
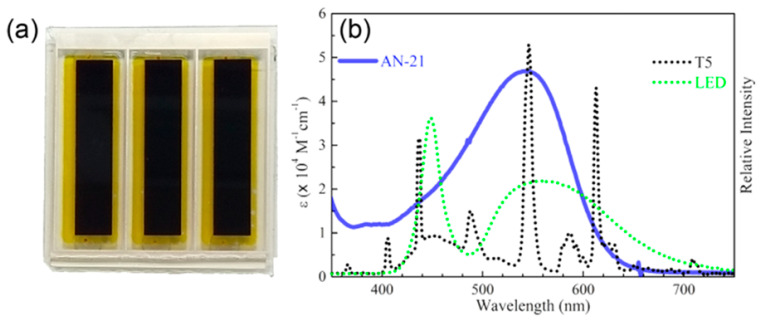
(**a**) Picture of a DSSC module built with the **AN-21** dye (active area: 9.12 cm^2^); (**b**) overlap of the UV-visible spectrum of **AN-21** (blue curve) and the emission spectra of T5 and LED Lights (dotted curves). Reproduced from Ref. [59] with permission from the American Chemical Society.

In 2020, the same group proposed further modifications of the **AN-11** structure, testing different anchoring groups and/or the elongation of the π-structure through the introduction of an additional ethynyl bridge [59]. Again, the **AN-21** dye (see Figure 13) was selected after a first screening under simulated AM 1.5 G irradiation for the realization of DSSC modules (active area = 9.12 cm^2^) and tested under dim-light irradiation. Like the previously reported molecules, **AN-21** was synthesized following a series of Sonogashira cross-coupling reactions, which allowed for keeping its estimated production cost below 60 USD/g.

Compared to **AN-3** and **AN-11**, the elongation of the π-structure in **AN-21** caused a further red-shift in the light absorption band, which was centered at 544 nm in THF solution and was well-matched with the emission spectrum of a common light source such as T5. The DSSC module efficiencies were further increased moving from **AN-11** to **AN-21** and PCE values among 12–14% and 9–13% were recorded under T5 and LED irradiation respectively (best PCE: 13.48% under T5 illumination at 1000 lux). Finally, the authors demonstrated that the **AN-21**-containing module had the ability to power indoor applications by introducing it inside a remote controller and showing a signal of response upon command after being put under 500 lux of T5 light for 10 min.

**Figure 14:**
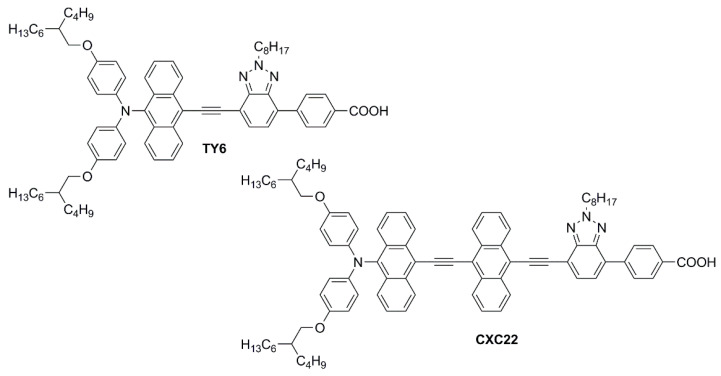
Structures of anthracene-based dyes with benzotriazole as the additional acceptor group [60,61].

Simultaneously, Wei, Yeh et al. investigated anthryl-based dyes containing an *N*,*N*-*bis*(*p*-alkyloxy-phenyl)amine as the donor, a benzotriazole as the additional acceptor group, and benzoic acid as the anchoring moiety [60,61]. The two photosensitizers (**TY6** and **CXC22**—Figure 14) differed in the elongation of the π-structure, with **CXC22** bearing an additional ethynyl-anthryl bridge in comparison to **TY6**. Both molecules were prepared by assembling the proper building blocks through the most common cross-coupling reactions (Sonogashira, Buchwald–Hartwig, and Suzuki–Miyaura) and exhibited three main absorption maxima in the UV-Vis region, with **CXC22** showing red-shifted (λ_max_ = 535 *vs.* 516 nm for **CXC22** and **TY6**, respectively) and more intense bands. Initially, **TY6** was employed as a DSSC photosensitizer in combination with an iodine-based electrolyte, either in the presence or in the absence of chenodeoxycholic acid (CDCA) as a co-adsorbent: the properties of the corresponding small-scale devices (active area = 0.25 cm^2^) were investigated under dim-light irradiation [60].

Using T5 as the light source, **TY6**-containing DSSCs exhibited remarkable PCE values ranging from 18.76 to 26.19% at variable illuminance levels (300–6000 lux) without CDCA, while the efficiencies rose to 21.40–28.56% in the presence of the co-adsorbent, especially thanks to a significant increase in terms of *V_oc_*. Under the same light source, DSSCs containing **N719** as the reference dye showed efficiencies varying between 12.00 and 18.91% (300–6000 lux). Once again, the improved performances registered with **TY6** compared to **N719** were attributed to the better matching between its light absorption profile and the emission spectrum of T5. Using a LED light source, the spectral matching with **TY6** was poorer and the PCE values were accordingly lower, but still higher than those obtained with **N719** (14.0–20.7% vs. 12.1–17.6%, respectively; illuminance: 300–6000 lux).

[Cu(dmp)_2_]^2+/+^(TFSI)_2/1_ redox couple (*E^0^* = +0.88 V vs. NHE) was then employed in both **TY6**- and **CXC22**-containing DSSCs [61]. Although the chosen light source was again T5, efficiencies of **TY6**-containing devices fell in the range of 14.56–21.34% (illuminance: 300–6000 lux), especially because of a significant loss in terms of *J_sc_*. However, under the same conditions, **CXC22** generated 71% higher photocurrents, which led to impressive PCE values ranging from 21.60% (300 lux) to 37.07% (6000 lux). Again, the increase in the photovoltaic performance was ascribed to the superior optical properties of **CXC22** in comparison to **TY6** and, consequently, to its better ability to harvest indoor light. At the same time, it should be pointed out that the very high PCEs achieved in this study were also due to excellent *V_oc_* values, which were reached thanks to the use of a copper-based redox mediator, having a much more positive redox potential compared to the traditional iodine-based one: a similar observation was made also in some studies dealing with the co-sensitization approach (see below, Section 3).

The long-term stability of dye **CXC22** was demonstrated by irradiating test cells containing I^−^/I_3_^−^ as the redox couple and 3-methoxypropionitrile (MPN) as the electrolyte solvent under one-sun illumination at 60 °C for 500 h: under these conditions, 96% of the initial PCE value was retained. Unfortunately, the stability test carried out on the **CXC22**-based DSSCs containing the [Cu(dmp)_2_]^2+/+^(TFSI)_2/1_ redox couple was ruinous, with only a 66% initial PCE value retained, even if the experimental conditions were milder (in the dark and at room temperature). Since the stability of the dye was already demonstrated, the reason for the failed test was ascribed to a possible reaction of the additive 4-*tert*-butylpyridine (4-TBP) with the copper complexes.

In conclusion, the anthracene core appears to be a smart choice for the elongation of the π-scaffold of organic D-π-A or D-A-π-A photosensitizers for indoor applications, thanks to its high thermal stability, good intramolecular charge transfer ability, and the possibility of obtaining high-responsive molecules in the entire visible region. Such advantageous optical properties can especially be achieved when the connection of the anthracenes with the other sections of the dyes occurs through C-C triple bonds, which keep the π-structure planar and increase the conjugation level. Finally, it must be highlighted that molecular diversity can be introduced quite easily on the 9- and 10-positions of the anthracene core thanks to the exploitation of the most common metal-catalyzed cross-coupling reactions and the commercial availability of dihalo-derivatives, such as 9,10-dibromoanthracene, which make the synthesis of anthryl-derivatives particularly affordable.

### 2.3. Benzopyrazine and Thienopyrazine-Based Dyes

**Figure 15:**
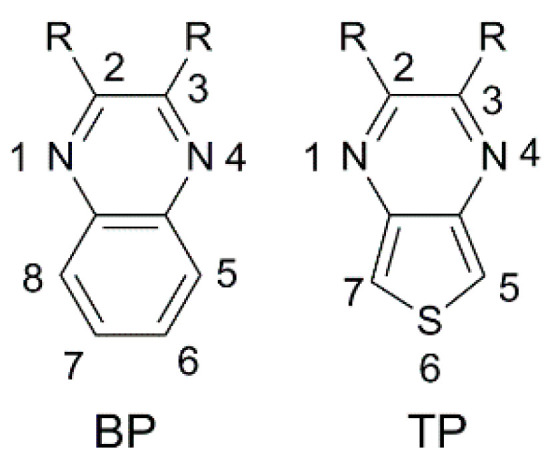
Benzopyrazine (**BP**) and thienopyrazine (**TP**) scaffolds with numbering of the ring positions.

Benzo [3,4-*b*]pyrazine (**BP**) is an electron-poor heterocyclic moiety (Figure 15), popularly used as the auxiliary acceptor in small band-gap polymeric materials with a D-A configuration [62]. For comparison, together with **BP**, a more electron-deficient entity, the thieno [3,4-*b*]pyrazine (**TP**), is often investigated.

**Figure 16:**
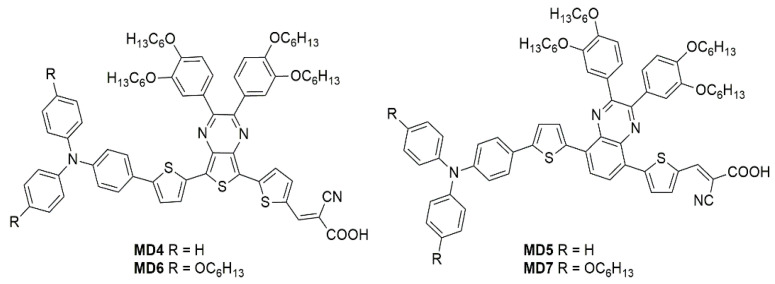
Benzopyrazine and thienopyrazine-based dyes [63].

The optical properties and solubility of the **BP**- and **TP**-molecules can be finely tuned introducing proper alkyl or aryl substituents on the 2,3-positions of the core. In this field, Diau and co-workers reported the synthesis and characterization of four new pyrazine-based dyes (**MD4-7**—Figure 16), which have been used in combination with I^−^/I_3_^−^ as the redox mediator to fabricate DSSCs and explore their photovoltaic properties both under 1 Sun irradiation and T5 fluorescent light at different illuminance levels, from 300 to 6000 lux [63].

**Figure 17:**
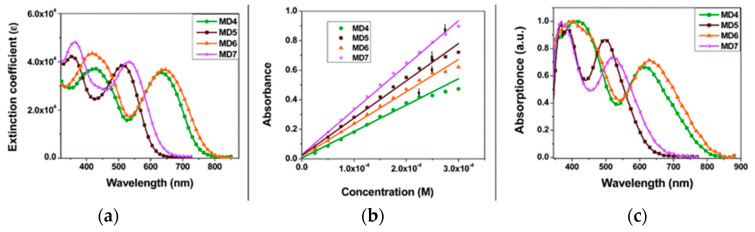
(**a**) Absorption spectra in THF solution (10 µM), (**b**) absorbance vs. concentration plot in THF, and (**c**) absorption spectra on TiO_2_ thin film for dyes **MD4-MD7**. Reproduced from Ref. [63] with permission from the Royal Society of Chemistry.

The synthetic pathway for **MD** dyes includes the functionalization of the corresponding fused-pyrazine cores through a Vilsmeier–Haack reaction, introducing an aldehyde moiety as the precursor of the final cyanoacrylic anchoring group, and a Stille–Migita cross-coupling to insert the two different donor groups. The thieno [3,4-*b*]pyrazine-based dyes, **MD4** and **MD6,** showed absorption maxima (*λ_max_*) both in the blue and red region of the visible spectrum: the lowest energy absorption peaks were located at 633 nm and 642 nm for **MD4** and **MD6**, respectively, with *λ_onset_* > 750 nm and molar attenuation coefficients still exceeding 10^4^ M^−1^ cm^−1^ at 700 nm (Figure 17a). On the other hand, the absorption maxima of **MD5** and **MD7** were located at 513 and 532 nm, respectively, with a blue-shift of around 110–120 nm compared to the **TP**-containing species: this behavior is coherent with the more electron-deficient nature of the thieno [3,4-*b*]pyrazine-core. As expected, increasing the donor strength of the electron-rich moiety by employing a *p*-alkyloxy-substituted triarylamine led to a red-shift of the absorption spectra of **MD6** and **MD7** in comparison to **MD4** and **MD5**. Solubility tests in THF solution did not indicate a relevant dye aggregation up to a concentration of 3 × 10^−4^ M, as shown by the good linearity of the absorbance vs. concentration plot (Figure 17b), but a slight dye aggregation was evident once adsorbed on 4 µm-thick TiO_2_ films (Figure 17c). Indeed, in all the spectra, the usual behavior of *H*-aggregates of the dyes adsorbed on titania, that is a red-shift of the *λ_onset_* and a blue-shift of the *λ_max_*, was recognizable for all the sensitizers, even though **MD4** and **MD6** seemed to have a higher tendency to aggregate than their **BP**-congeners.

For these reasons, CDCA (1 mM) was used as the co-adsorbent and added during dye soaking to minimize the aggregation of the dyes and to passivate the TiO_2_ surface to suppress the dark current.

Thanks to their almost panchromatic absorption in the visible region, the **MD**-dyes demonstrated a good spectral matching with the emission spectrum of the T5 fluorescent lamp. Both **BP**-based dyes (**MD5** and **MD7**) showed PCE values higher than 15% even under only 300 lux illumination, and **MD7** exhibited the highest efficiency among all the tested sensitizers, reaching 27.17% PCE under 6000 lux irradiance, which is comparable with the efficiency of the **N719**-based cells fabricated and measured under similar conditions (27.64%).

**Figure 18:**
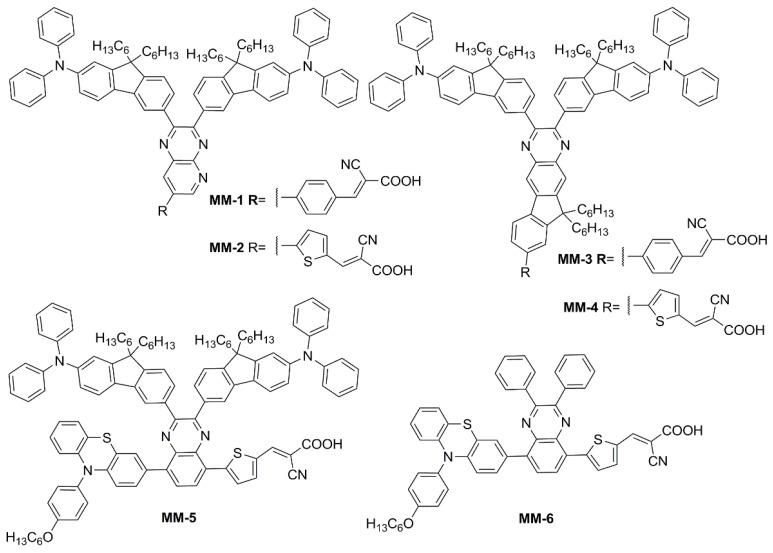
Electron-rich benzopyrazine-based dyes **MM1**–**6** [64].

Subsequently, in 2019 Chang et al. proposed a series of Y-shaped D-A-π-A dyes containing **BP** and **BP**-like moieties. Six metal-free organic dyes (**MM-1**–**6**, Figure 18) were synthesized through stepwise modifications on the **BP** structure by introducing electron-donating substituents at different positions of the **BP** moiety [64].

**Figure 19:**
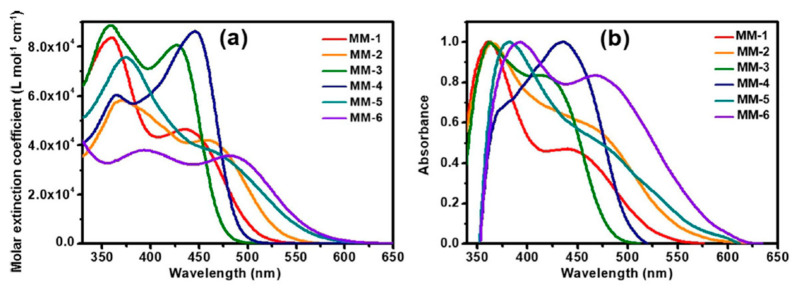
Absorption spectra of the **MM**-series dyes (**a**) in THF solution (0.3 mM) and (**b**) adsorbed on a thin TiO_2_ film. Reproduced from Ref. [65] with permission from Wiley.

The synthesis of compounds **MM-1**–**6** involved an initial condensation reaction between a diketone precursor and the respective diamine to form the **BP** unit; subsequently, the other building blocks were assembled via Sonogashira, Stille–Migita, and Suzuki cross-couplings. All the **MM**-dyes exhibited two broad and intense light absorption peaks in the 359–484 nm range. (Figure 19a). Compared with the phenylene π-spacers in **MM-1** and **MM-3**, the introduction of a thiophene ring in **MM-2** and **MM-4** induced a higher planarity of the system, extending its π-conjugation and, therefore, causing a red-shift of the absorption maxima by approximately 18–22 nm. Even more red-shifted peaks were observed for the dyes **MM-5** and **MM-6**, thanks to the introduction of a phenothiazine-based donor group into their structures. Spectra of the dyes adsorbed on a mesoporous TiO_2_ film appeared quite similar to those in solutions (Figure 19b). A slight red-shift in light absorption was observed for dyes **MM-1** and **MM-2**, which was ascribed to a possible *J*-aggregation of the molecules in the solid state, while H-aggregation and/or deprotonation of the cyanoacrylic moiety could justify the blue-shift of the *λ_max_* for **MM-4** and **MM-6**.

DSSC prepared with the **MM**-dyes and I^−^/I_3_^−^ as the redox mediator showed promising power conversion efficiencies (PCEs) under 1 Sun illumination: in particular, the dyes carrying a donor group (a phenothiazine) at 5 or 8-position of **BP** (**MM-5** and **MM-6**) displayed higher photocurrents and PCEs than those containing the donors only at 2,3-positions (**MM-1**–**4**). The authors also explored the possibility of using these dyes under the illumination of a TL84 fluorescent lamp in the intensity range of 600–2500 lux. The devices containing the sensitizers **MM-1**–**4** showed unremarkable PCE values up to 13% even under a stronger illumination of 2500 lux, possibly due to their narrower absorption bandwidths; on the other hand, the two dyes **MM-5** and **MM-6** displayed PCE values higher than 18% under 1000 lux. **MM-6** showed the best performance among all the sensitizers, reaching a PCE of 28.95% (2500 lux) when using deoxycholic acid as a co-absorbent. The authors further looked into the effect of co-adsorption, mixing **MM-3** and **MM-6** at different ratios, to get photoanodes with broader light absorption and produce higher currents. **MM-3** and **MM-6** were subsequently deposited on TiO_2_, finding that the optimized deposition times for **MM-3** and **MM-6** were 4 and 8 h, respectively. The best PCE was 30.45% under TL84 fluorescent indoor light source at 2500 lux. A stability test of the devices was carried out at 25 °C under both TL84 indoor light and AM 1.5 G in ambient conditions. The device containing both **MM-3** and **MM-6** retained 80–82% of the initial efficiency at every illuminance (600–2500 lux) after 168 h, indicating good stability in these conditions.

**Figure 20:**
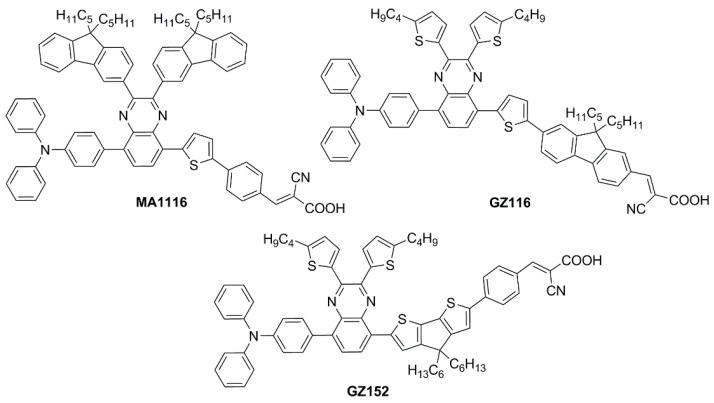
Structures of benzopyrazine-based dyes with extended conjugation [65,66].

Sun et al. in 2021 described a series of new organic **BP**-based dyes with a D-A-π-A scaffold integrated with fluorenyl units, which were used to extend the conjugation of the π-spacer or for the decoration of the 2 and 3 positions of the **BP** scaffold (Figure 20) [65]. The synthesis of the dyes (**GZ** and **MA** series) was carried out through a series of Miyaura borylation and Suzuki–Miyaura coupling procedures and concluded with a Knoevenagel condensation. Among all the reported structures, the broader absorption bands in the visible region of **GZ116** and **MA1116** (Figure 20) compared to other dyes made these two molecules promising candidates for indoor applications. In *N*,*N*-DMF solution, indeed, these dyes exhibited two or more main absorption peaks in the range of 335–600 nm: furthermore, both molecules showed almost panchromatic light-harvesting properties, with the absorption edges approaching 600 nm.

Thus, solar cells containing **GZ116** and **MA116** as the photosensitizers and I^–^/I_3_^–^ as the redox mediator were investigated under indoor conditions using a T5 fluorescent lamp with different light intensities, between 300 and 6000 lux. The devices fabricated with **MA116** displayed a high PCE of 26.81% (*J_sc_* = 0.93 mA cm^–2^, *V_oc_* = 0.68 V, and *FF* = 0.76), while **GZ116** displayed a PCE of 25.38% (*J_sc_* = 0.88 mA cm^–2^, *V_oc_* = 0.69 V, and *FF* = 0.76) at 6000 lux illumination. Both dyes exhibited a comparable performance to the standard organometallic photosensitizer **N719**, whose cells were fabricated and measured under the same conditions. These studies demonstrated that placing a fluorenyl unit on the electron-poor quinoxaline scaffold could be a promising approach for boosting both the photocurrent and the photovoltage of DSSC devices for outdoor and indoor applications.

Later, the same group studied a new dye named **GZ152** (Figure 20) to assess the effect of a longer π-conjugation in sensitizers containing a 2,3-*bis*(5-butylthiophen-2-yl)quinoxaline as an auxiliary acceptor unit [66]. The cyclopentadithiophene (CPDT) unit was introduced as an extended π-conjugated section to further modulate the optical properties of **GZ152**, which was tested in DSSCs using an I^−^/I_3_^−^-based electrolyte under simulated AM 1.5 G light irradiation, giving a maximum PCE of 8.61%. Once adsorbed on TiO_2_, the dye displayed a broad, almost panchromatic light absorption in the visible region, which was well-matched with the emission of the T5 fluorescent lamp. Thus, the performance of **GZ152**-containing DSSCs was further evaluated under T5 illumination with variable light intensity from 500 to 6000 lux. As expected, the PCE values increased with the illumination intensity, and the best performance was achieved at 6000 lux, with a PCE of 28.31% (*J_sc_* = 1.15 mA cm^−2^, *V_oc_* = 0.62 V, and *FF* = 0.77).

In summary, **BP** and **TP** units can be used as auxiliary acceptors in the conjugated spacer of D-π-A or D-A-π-A dyes for indoor applications thanks to a beneficial red-shift of the absorption spectra, especially ascribed to a deepening of the LUMO energy level, and an improved photostability. The functionalization of these heterocyclic cores can be easily tuned by following robust synthetic strategies based on the most common cross-coupling reactions allowing for the introduction of the various different building blocks.

### 2.4. Miscellaneous Dyes

**Figure 21:**
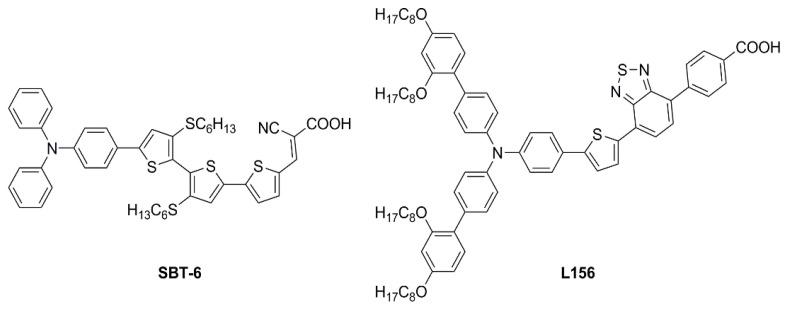
Structures of dithioalkylbithiophene dyes **SBT-6** and **L156** [67,68].

In the race toward increasing performances, several other scaffolds have been the subject of individual studies, leading to the characterization of a variety of photochemical properties and enriching the dye library for low-light environment conditions. In 2020, Ho’s group focused on some dithioalkylbithiophene (SBT)-based chromophores (Figure 21) to investigate the effect of extending π-bridge conjugation and introducing different S-alkyl chains onto the molecules’ backbones [67]. The strong S(Thio)-S(R) intramolecular interaction appeared to increase the planarity of the molecules, broadening their absorption spectra (*λ_onset_* > 600 nm) and leading to high *J_SC_* values in full Sun photovoltaic characterization (AM 1.5 G, 100 mW cm^−2^). Due to the obvious proneness to aggregation of such compounds, the authors also performed an extensive study on the addition of the nonconductive co-adsorbent CDCA in combination with the sensitizers. Thanks to the addition of CDCA (10 mM) the best-performing **SBT-6**-based device exhibited a remarkably improved efficiency of up to 9.47% with the I^−^/I_3_^−^ couple as the electrolyte under 1 Sun illumination. The latter configuration was then tested under a T5 light source and compared with the standard **N719** dye under increasing light intensity (from 900 to 6000 lux). A good PCE of 23.57% was recorded under 6000 lux illumination, higher than the performance reported for **N719**.

In the same year, Ferdowsi and co-authors presented a work describing the novel dye **L156** (Figure 21), containing a triphenylamine (TPA) segment and 4-(benzo[*c*][1,2,5]thiadiazol-4-yl)benzoic acid as electron-rich and -deficient moieties, respectively, connected by a thiophene π bridge [68]. Following an extensive optimization of the DSSC devices setup under simulated AM 1.5 G sunlight, the sensitizer was eventually tested under indoor-light conditions both at 200 lux and 1000 lux. Samples were prepared by using 30NR-D (dispersive titania paste) with 30 nm average particle size, and different concentrations of [Cu(tmby)_2_]^2+/1+^ (tmby, 4,4′,6,6′-tetramethyl-2,2′-bipyridine; 0.06, 0.03, 0.01 M Cu^2+^) as the redox mediator. While the performances reported at 200 lux were generally low, the best efficiencies were achieved for devices featuring the lowest Cu^2+^ concentration (0.01 M) under 1000 lux of illumination, reaching the interesting result of 21.9% PCE and an output power density (*P_out_*) of 67.3 µW cm^−2^.

**Figure 22:**
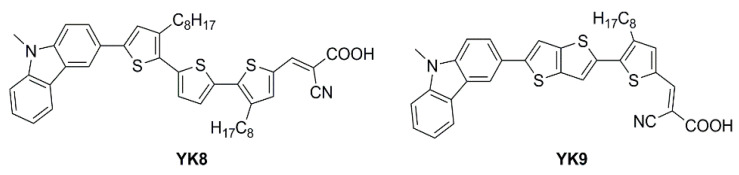
Structures of carbazole-based D–π–A dyes **YK8** and **YK9** [69].

An extensive study was presented in 2021 by Haridas and co-workers reporting two new molecularly engineered, cost-effective, metal-free, carbazole-based D–π–A sensitizers (**YK8** and **YK9**—Figure 22) containing two different π-spacers, suitable for indoor photovoltaic applications [69].

**Figure 23:**
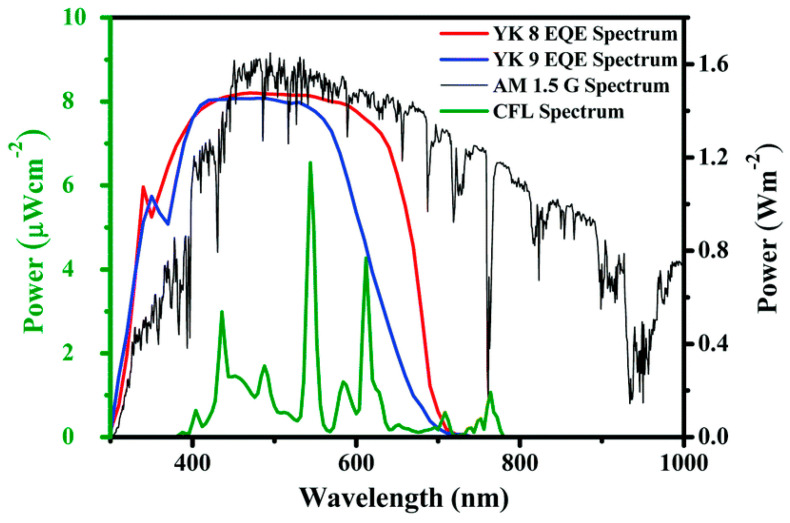
Overlap of Osram T2 fluorescent lamp and AM 1.5 G power density spectra with the *EQE* spectra of DSSCs built with dyes **YK8** and **YK9**. Reproduced from Ref. [69] with permission of the Royal Society of Chemistry.

The **YK8** sensitizer was endowed with bis-octyl chains on a terthiophene core to reduce aggregation and prevent charge carrier recombination, potentially yielding a better electron lifetime, whereas the **YK9** dye was designed and synthesized using a more planar thienothiophene linker for better donor–acceptor interactions, facilitating rapid electron migration to the TiO_2_ layer. The *J*/*V* measurements of the corresponding DSSCs were conducted under Osram 14 W T2 cool daylight fluorescent tube illumination at various intensities (700, 1000, 1500, and 2000 lux). A conventional iodide/triiodide electrolyte was exploited for all the experiments. As commonly reported, the authors observed an increase of *J_SC_* and *V_OC_* for both **YK8** and **YK9** dyes as the light intensity increased, reaching an impressive maximum power conversion efficiency of 30.24% ± 1.23% for **YK8** at 1500 lux, while the DSSCs employing **YK9** showed a maximum PCE of 20.11% ± 0.96% at 2000 lux. As expected, compared to **YK9**, the lower aggregation on the surface of TiO_2_ and the reduction of charge recombination phenomena due to the larger number of alkyl chains allowed **YK8**-containing cells to achieve an improved electron lifetime, leading to a better open-circuit voltage. In addition, the extended π linker led to a better electron diffusion length contributing to superior charge collection efficiency. Further, the red-shifted absorption in the 550–700 nm range for the **YK8** sensitizer matches the spectra of the cool daylight fluorescent tube used for illumination, leading to better light harvesting, as shown by the corresponding *EQE* spectra (Figure 23). Finally, **YK8**-based cells presented a higher shunt resistance at 1500 lux, which may have also contributed to its better PCE at this particular light intensity. Interestingly, Haridas’ group showed a custom-designed self-powered temperature sensor with an LCD that drew power from the indoor solar cells containing the **YK8** sensitizer. Four solar cells, each having an active area of 0.31 cm^2^ were serially interconnected (net active area of 1.24 cm^2^) to successfully power the temperature sensor under an illumination of 500 lux.

**Figure 24:**
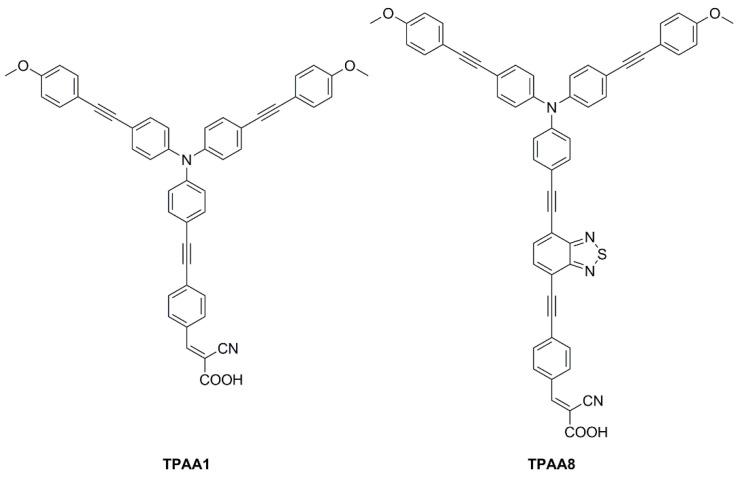
Structures of D-π-A propeller-shaped dyes **TPAA1** and **TPAA8** [70,71].

In 2021, Yoosuf et al. expanded their previous work on D-π-A propeller-shaped dyes (**TPAA1**—Figure 24) [70] by synthesizing and testing a new molecularly engineered D-A-π-A triphenylamine dye featuring conjugated triple bonds and a benzothiadiazole unit as auxiliary acceptor (**TPAA8**—Figure 24) [71]. Photovoltaic performances of cells built with **TPAA1** and **TPAA8** sensitizers were measured under 1000 lux illumination intensity using a warm white LED light, employing both I^–^/I_3_^–^ and [Co(bpy)_3_]^2+/3+^ as redox couples. The best performance under indoor light was showcased by **TPAA8**, delivering a maximum power conversion efficiency of 11.03% along with a [Co(bpy)_3_]^2+/3+^ redox mediator, while 8.74% was achieved when using an I^−^/I_3_^−^ redox mediator. For comparison, **TPAA1** exhibited very low power conversion efficiencies of 0.30% and 2.50% along with [Co(bpy)_3_]^2+/3+^ and the I^−^/I_3_^−^ electrolyte, respectively.

**Figure 25:**
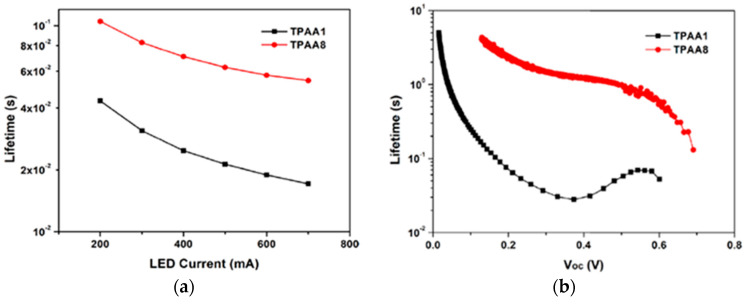
(**a**) Electron lifetime as a function of LED current obtained from TVD; (**b**) lifetime as a function of *V_oc_* obtained from OCVD. Reproduced from Ref. [71] with permission from Elsevier.

Transient photovoltage decay (TVD, Figure 25a) and open circuit photovoltage decay (OCVD, Figure 25b) experiments suggested a largely increased electron lifetime and a slower recombination rate linked with the introduction of the additional benzothiadiazole auxiliary acceptor in **TPAA8**, which helped to maintain reasonably good open-circuit potential and fill factor, contributing to the improved photovoltaic performances of that dye.

**Figure 26:**
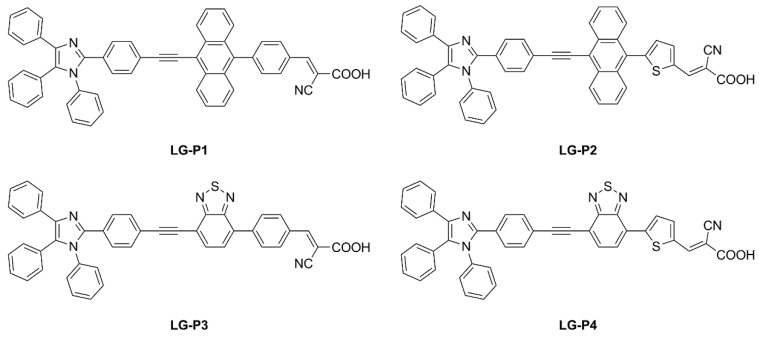
Structures of triphenylimidazole-based dyes **LG-P1**–**4** [72].

Giribabu et al. recently published three articles detailing their extensive work on triphenylimidazole-based metal-free organic dyes. In these works, four sensitizers were synthesized through a series of optimized Sonogashira cross-coupling reactions and then tested in combination with copper-based electrolytes [72,73], as well as with the standard I^−^/I_3_^−^ redox couple [74]. Firstly, different structures of D-D-π-A (**LG-P1**—Figure 26) and D-A-π-A (**LG-P3**—Figure 26) dyes were investigated, bearing anthracene and benzothiadiazole as the auxiliary donor and acceptor units, respectively [72]. The dyes were designed to have a relatively positive ground state oxidation potential to make them compatible with copper electrolyte [Cu(dmp)_2_]^2+/1+^. Nonetheless, efficiencies under 1000 lux daylight LED illumination were low, mainly limited by strong recombination processes. In particular, such processes were more intense on the **LG-P1** sensitizer, leading to PCE values of 0.13%, while **LG-P3**-based devices showed a 9.14% efficiency.

Charge extraction (CE), open-circuit voltage decay (OCVD), and electrochemical impedance spectroscopy (EIS) experiments confirmed that the more planar geometry and better conjugation of the D–A–π–A **LG-P3** dye contributed to its better photon absorption, injection, and charge collection efficiencies, resulting in an improved IPCE profile and *Jsc* values. Interestingly, a more recent work from the same group expanded the study to dyes **LG-P2** and **LG-P4** (Figure 26). The latter were similar to sensitizers **LG-P1** and **LG-P3,** respectively, but bore a thiophene group as a π linker instead of benzene, which was supposed to guarantee better orbital overlap between the adjacent donor/acceptor groups and the π spacer thanks to their lower dihedral angles [73]. In contrast to the previous report, the device employing the **LG-P2** dye having anthracene as the auxiliary donor in a D-D-π-A architecture displayed a better PCE of 7.46%, while **LG-P4**, featuring the benzothiadiazole unit, led to an efficiency of 0.97%. Again, strong recombination processes are linked to the lower injection driving force, hampering the performance of **LG-P4** dye.

More recently, in 2022 Giribabu et al. expanded their studies by employing the aforementioned molecules in I^−^/I_3_^−^ electrolyte-based DSSC devices [74]. Under the very same indoor illumination conditions used in the previous papers (1000 lux daylight LED), sensitizer **LG-P1** resulted in the best-performing compound with a PCE of 10.53%. Interestingly, in tests performed using a fluorescent lamp (1000 lux daylight CFL), dye **LG-P2** showed the top efficiency among the novel sensitizers, reaching a PCE of 9.19%.

**Figure 27:**
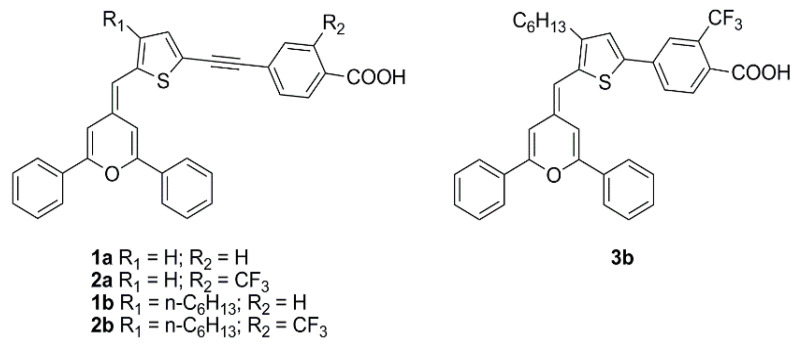
Structures of DSSC dyes containing the pyranylidene scaffold [75].

The pyranylidene scaffold has been used by Orduna and coworkers as donor moiety for a series of small sensitizers in which insertion of triple bond and/or trifluoromethyl groups in the π-bridge was studied (Figure 27) [75]. Test cells sensitized with CH_2_Cl_2_ solutions containing 0.1 mM conc. of the dye and 0.3 mM of anti-aggregating additive CDCA were first analyzed under standard AM 1.5 G illumination using I^−^/I_3_^−^ redox couple as the electrolyte. Compound **2b** achieved the best performances within the series, demonstrating a positive influence of the acetylenic bond in the molecule backbone due to the increased electron transfer between the donor and acceptor units. Moreover, the presence of alkyl chains limited the aggregation (compounds **1b** and **2b**), while the incorporation of a CF_3_ group in the benzene ring (**2a-b**) increased the *V_OC_* values, improving the efficiencies. EIS experiments seemed to confirm that the alkyl substituent and the CF_3_ group reduce the recombination processes. Tests carried out in lower light conditions (77 mW cm^−2^) or with a fluorescent lamp (OSRAM 930/18W) at 2000 lux (0.708 mW cm^−2^) and 1000 lux (0.350 mW cm^−2^) showed a similar trend. The highest values were obtained at 1000 lux for compound **2b** reaching a η of 5.64%, with *J_SC_* of 59.7 µA cm^−2^, *V_OC_* of 445 mV, and *FF* of 0.74.

**Figure 28:**
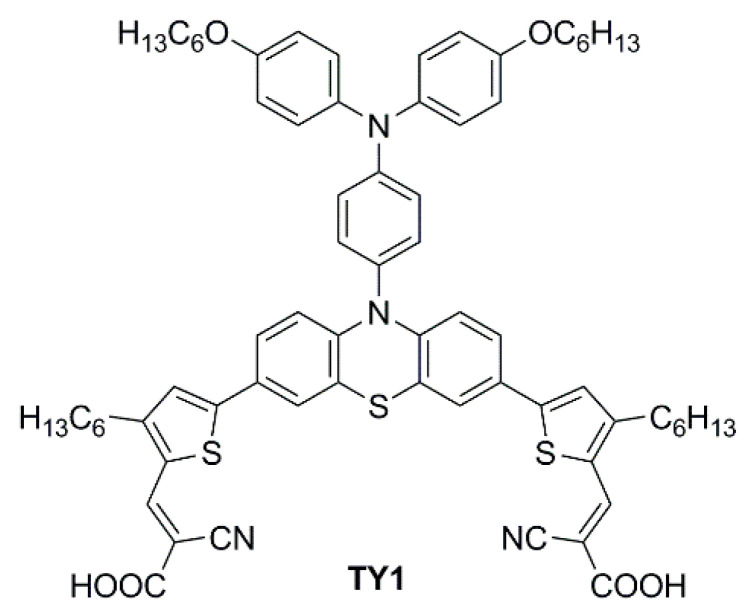
Structure of the double-anchored phenothiazine dye **TY1** [76].

In 2023, Li et al. reported a series of sensitizers bearing a double-anchored phenothiazine scaffold, arranged in a D(-π-A)_2_ skeleton [76]. A preliminary photovoltaic study was conducted under simulated AM 1.5 G illumination using I^−^/I_3_^−^ as a redox mediator. The best-performing dye was **TY1** (Figure 28), whose cells showed a good PCE of 8.33%, with an open-circuit voltage (*V_OC_*) of 0.72 V, a short-circuit current density (*J_SC_*) of 16.19 mA cm^−2^, and a fill factor (*FF*) of 0.71. The authors also performed an extensive study on the addition of the nonconductive co-adsorbent CDCA in combination with **TY1**, finding that a high concentration of CDCA (CDCA/**TY1** ≈ 33/1) is necessary to successfully occupy the TiO_2_ surface not covered by the dye, decreasing the aggregation between dye molecules and leading to enhanced *J_SC_*.

Finally, **TY1** was tested in indoor photovoltaic conditions using a halogen lamp (Philips TL5 lamp, 14 W, 6500 K), under different light intensities. The optimal **TY1**/CDCA-based cell reached good efficiencies of 21.16% at 1000 lux (office environment), 19.53% at 600 lux (conference hall), and 16.75% at 300 lux (living room). Moreover, a durability test was also performed exhibiting a decent long-term stability within 20 days.

Materials used, essential experimental details, and photovoltaic properties of the devices prepared with the dyes discussed in this chapter are reported in Table 3.

### 2.5. Novel Organic Sensitizers for Indoor DSSCs: Conclusions

Despite the large number of dyes recently reported for indoor DSSC applications, a clear development path for organic structures specially designed for dim-light harvesting appears difficult to identify, except when a number of consecutive studies were conducted on specific dye classes by the same research groups. Nevertheless, the recurrence of some building blocks, such as fluorene, anthracene, and benzopyrazine, allows for drafting of some potential guidelines to be followed for the design of such kinds of sensitizers:

1. D-A-π-A structure: despite some exotic configurations that were tested over the years, the most reliable design for DSSC dyes is still the D-A-π-A structure, regardless of whether the energy source is the Sun or artificial light (e.g., dyes **CXC22** [61] and **GZ152** [66]). This configuration usually ensures intense and broad light absorption bands, well-aligned HOMO/LUMO energy levels, and long-term stability.
2. Matching with the light source: in most cases, the DSSCs that show the best PCE values under indoor conditions are those that contain sensitizers featuring either a light absorption spectrum well-matched to the emission spectrum of the light source or a panchromatic absorption in the visible region (e.g., **AN21** [59] and **YK8** [69]). A careful design of the structure of the dye is mandatory to obtain the desired dye optical properties.
3. Bulkiness: the introduction of bulky alkyl or (hetero)aryl groups into the dye structures (e.g., **RY3** [52] and **MD5,7** [63]) was revealed to be beneficial for reducing undesired aggregation on the semiconductor and shielding the TiO_2_ layer from the electrolyte, thus limiting recombination.

From the synthetic point of view, the best-performing dyes are usually prepared by exploiting the most common metal-catalyzed cross-coupling processes (e.g., Suzuki–Miyaura and Stille–Migita reactions), which are consolidated organic transformations, potentially scalable for an industrial application. Furthermore, in the perspective of increasing sustainability and limiting the costs of potential scaled-up synthesis, more attention should be paid to the design of greener processes, for example by avoiding the use of toxic and flammable solvents and/or reducing the employment of organometallic species through the exploitation of procedures based on C-H activation reactions [77,78].

To conclude, we can state that an enormous number of new individual dye structures are theoretically still available for DSSC indoor applications. To limit the chemical space to cover, however, future research in this field should focus on finding the perfect match between the dye light absorption properties and the emission spectrum of the light source used in each specific application, differently from the outdoor conditions where the only light source is the Sun. Furthermore, greater efforts should be made to design dyes having the right energy level alignment to work with organometallic redox mediators, like Co- and Cu-based molecules, since they have not been thoroughly explored so far in this field.

Besides the development of rationally designed individual dye structures, alternative approaches to improve the light-harvesting properties of the cells, such as the co-sensitization strategy or the application of covalent companion dyes, have also been explored, which will be the subject of the following paragraphs.

**Table 3:** Materials used and photovoltaic properties of the devices prepared with the organic dyes discussed in this review.

Dye	*λ_abs_* (nm) *ε* (M^−1^/cm^−1^)	Semi Conductor Layer	Additives	Electrolyte	Counter Electrode	Active Area (cm^2^)	Lamp Type ^(a)^	Illuminance (Lux) ^(b)^	PCE (%)	Ref.
**TKU-1**	441 (27,400) (THF)	TiO_2_ 10–12 μm (transp) 5 μm scatt. layer	none	I^−^/I_3_^−^ (EL 201, Everlight Taiwan)	Platinum	0.28 (mask)	T5 Fluorescent	600	7.80	[36]
							Planar White LED		6.42	
**TKU-2**	463 (27,100) (THF)						T5 Fluorescent		12.73	
							Planar White LED		11.71	
**TKU-3**	441 (30,500) (THF)						T5 Fluorescent		9.99	
							Planar White LED		6.56	
**TKU-4**	470 (32,700) (THF)						T5 Fluorescent		13.43	
							Planar White LED		11.49	
**TKU-5**	432 (22,000) (THF)						T5 Fluorescent		12.00	
							Planar White LED		10.76	
**TKU-6**	480 (33,900) (THF)						T5 Fluorescent		12.74	
							Planar White LED		12.21	
**OMS4**	364 (39,700) THF	TiO_2_ 12 μm (transp.) 6 μm scatt. Layer	CDCA (10 mM)	I^−^/I_3_^−^ TBP	Platinum	0.28 (mask)	D65	1000	2.48	[37]
							CWF	1000	7.26	
								2200	8.12	
								600	2.40	
							TL84	1000	7.59	
								2500	8.78	
**RY1**	440 (43,400) CH_2_Cl_2_	TiO_2_ 12 μm (transp.) 6 μm scatt. layer	CDCA (10 mM)	I^−^/I_3_^−^ TBP	Platinum	0.28 (mask)	TL84 (4100 K)	1000 (0.185)	16.64 ± 1.38	[38]
								2500 (0.462)	17.94 ± 0.91	
							CWF (4150 K)	1000 (0.185)	14.32 ± 1.36	
								2500 (0.462)	16.06 ± 0.77	
**RY2**	397 (41,700) CH_2_Cl_2_						TL84 (4100 K)	1000 (0.185)	8.23 ± 0.62	
								2500 (0.462)	9.02 ± 0.40	
**RY3**	450 (45,000) CH_2_Cl_2_						TL84 (4100 K)	1000 (0.185)	18.15 ± 1.00	
								2500 (0.462)	20.83 ± 0.84	
							CWF (4150 K)	1000 (0.185)	16.11 ± 1.09	
								2500 (0.462)	17.49 ± 0.79	
**RY4**	410 (37,700) CH_2_Cl_2_						TL84 (4100 K)	1000 (0.185)	10.03 ± 0.62	
								2500 (0.462)	11.76 ± 0.50	
**RY5**	444 (45,100) CH_2_Cl_2_						TL84 (4100 K)	1000 (0.185)	16.94 ± 1.12	
								2500 (0.462)	19.74 ± 0.76	
							CWF (4150 K)	1000 (0.185)	15.93 ± 1.06	
								2500 (0.462)	18.29 ± 0.70	
**RY6**	402 (28,300) CH_2_Cl_2_						TL84 (4100 K)	1000 (0.185)	9.36 ± 1.32	
								2500 (0.462)	11.74 ± 0.83	
**AN-3**	499 (46,900) THF	TiO_2_ 12 μm (opaque)	none	I^−^/I_3_^−^ PMII, TBP	Platinum	36	T5	200 (0.065)	3.11 ± 0.332	[41]
								600 (0.187)	4.94 ± 0.15	
								1000 (0.336)	5.45 ± 0.09	
							T8	200 (0.065)	2.08 ± 0.126	
								600 (0.200)	3.59 ± 0.12	
								1000 (0.338)	4.85 ± 0.09	
							LED	200 (0.065)	2.14 ± 0.219	
								600 (0.186)	3.59 ± 0.14	
								1000 (0.313)	4.94 ± 0.11	
**AN-11**	528 (46,100) THF	TiO_2_ 9 μm (translucent) 6 μm scatt. layer	none	I^−^/I_3_^−^ PMII TBP	Platinum	0.25	T5	1000 (0.34)	10.53 ± 0.54	[43]
							LED	1000 (0.31)	10.18 ± 0.46	
						26.80 (rigid)	T5	200	9.08 ± 0.11	
								600	11.17 ± 0.18	
								1000 (0.34)	11.94 ± 0.16	
							LED	200	9.68 ± 0.13	
								600	10.95 ± 0.17	
								1000 (0.31)	11.26 ± 0.21	
						19.80 (flexible)	T5	200	8.15 ± 0.15	
								600	9.26 ± 0.19	
								1000 (0.34)	9.60 ± 0.16	
							LED	200	8.08 ± 0.18	
								600	9.37 ± 0.19	
								1000 (0.31)	9.51 ± 0.25	
**AN-12**	433 (16,800) 515 (19,900) THF					0.25	T5	1000 (0.34)	6.08 ± 0.55	
							LED	1000 (0.31)	5.33 ± 0.40	
**AN-14**	438 (21,000) 517 (2.34) THF					0.25	T5	1000 (0.34)	5.80 ± 0.35	
							LED	1000 (0.31)	5.32 ± 0.24	
**AN-21**	316 (42,000) 544 (46,900) THF	TiO_2_ 8 μm (translucent) 2 μm scatt. Layer	none	I^−^/I_3_^−^ TBP	Platinum	9.12	T5	200	11.77 ± 0.48	[44]
								600	13.30 ± 0.32	
								1000	13.48 ± 0.53	
							LED	200	9.13 ± 0.40	
								600	11.29 ± 0.3	
								1000	12.82 ± 0.18	
**TY6**	419 (25,000) 516 (16,000) THF	TiO_2_ 12 μm (transp.) 6 μm scatt. layer	none	I^−^/I_3_^−^ PMII TBP	Platinum	0.36 (mask)	T5	300 (0.085)	18.76	[45]
								600 (0.177)	19.93	
								900 (0.348)	21.85	
								1200 (0.517)	22.80	
								2400 (0.703)	24.06	
								3600 (1.05)	25.03	
								4800 (1.40)	25.68	
								6000 (1.74)	26.19	
			CDCA (0.3 mM)					350 (0.104)	21.40	
								600 (0.177)	21.88	
								900 (0.348)	23.64	
								1200 (0.517)	24.43	
								2400 (0.703)	25.67	
								3600 (1.05)	26.88	
								4800 (1.40)	27.66	
								6000 (1.74)	28.56	
							LED	350 (0.11)	13.997 ± 0.388	
								600 (0.19)	15.383 ± 0.361	
								900 (0.29)	16.211 ± 0.344	
								1200 (0.38)	16.966 ± 0.284	
								2400 (0.77)	18.728 ± 0.445	
								3600 (1.15)	19.527 ± 0.475	
								4800 (1.54)	20.208 ± 0.563	
								6000 (1.9)	20.718 ± 0.581	
**TY6**	419 (25,000) 516 (16,000) THF	TiO_2_ 4 μm meso- porous	CDCA (0.3 mM)	Cu(dmp)_2_ (TFSI)_1/2_ LiTFSI TBP	poly-N-vinyl-2-pyrrolidone-capped Pt nanoclusters (PVP-Pt)	0.16	T5	300 (0.095)	12.26 ± 1.77	[46]
								600 (0.19)	14.05 ± 1.22	
								900 (0.29)	15.43 ± 0.89	
								1200 (0.39)	16.17 ± 0.91	
								2400 (0.78)	18.15 ± 0.82	
								3600 (1.15)	19.22 ± 0.66	
								4800 (1.55)	19.81 ± 0.78	
								6000 (1.9)	20.87 ± 0.83	
**CXC22**	487 (37,000) 535 (27,000) THF							300 (0.095)	20.89 ± 0.91	
								600 (0.19)	23.48 ± 0.64	
								900 (0.29)	24.56 ± 0.62	
								1200 (0.39)	25.65 ± 0.75	
								2400 (0.78)	28.87 ± 0.90	
								3600 (1.15)	31.60 ± 1.11	
								4800 (1.55)	34.21 ± 1.36	
								6000 (1.9)	35.66 ± 1.32	
**MD4**	427 (33,832) 633 (32,231) THF	TiO_2_ 7 μm (transp.) 5 μm scatt. layer	CDCA (1 mM)	I^−^/I_3_^−^ DMPII TBP	Platinum	0.16	T5	300 (0.09)	6.39 ± 0.13	[48]
								600 (0.18)	6.67 ± 0.11	
								900 (0.27)	6.81 ± 0.19	
								6000 (1.7)	8.62 ± 0.16	
**MD5**	356 (41,667) 513 (37,998) THF							300 (0.09)	15.1 ± 0.67	
								600 (0.18)	16.49 ± 0.4	
								900 (0.27)	17.38 ± 0.38	
								6000 (1.7)	23.17 ± 0.22	
**MD6**	420 (40,188) 642 (33,530) THF							300 (0.09)	12.08 ± 0.02	
								600 (0.18)	12.79 ± 0.05	
								900 (0.27)	13.24 ± 0.11	
								6000 (1.7)	16.86 ± 0.23	
**MD7**	365 (47,545) 532 (39,360) THF							300 (0.09)	18.95 ± 0.69	
								600 (0.18)	20.16 ± 1.10	
								900 (0.27)	21.10 ± 1.20	
								6000 (1.7)	27.17 ± 1.44	
**MM-1**	435 (46,300) CH_2_Cl_2_	TiO_2_ 6 μm (transp.) 6 μm scatt. layer	none	I^−^/I_3_^−^ TBP	Platinum	0.28	TL84	600 (0.110)	4.43 ± 0.41	[49]
								1000 (0.185)	7.50 ± 0.97	
								2500 (0.462)	7.72 ± 0.59	
**MM-2**	457 (42,000) CH_2_Cl_2_							600 (0.110)	6.71 ± 0.61	
								1000 (0.185)	8.52 ± 1.08	
								2500 (0.462)	9.03 ± 0.67	
**MM-3**	427 (80,600) CH_2_Cl_2_							600 (0.110)	7.35 ± 0.57	
								1000 (0.185)	9.64 ± 0.56	
								2500 (0.462)	9.86 ± 0.64	
**MM-4**	445 (86,200) CH_2_Cl_2_							600 (0.110)	10.16 ± 0.67	
								1000 (0.185)	11.07 ± 0.35	
								2500 (0.462)	12.14 ± 0.63	
**MM-5**	470 (36,500) CH_2_Cl_2_							600 (0.110)	12.38 ± 0.86	
								1000 (0.185)	18.99 ± 1.09	
								2500 (0.462)	19.89 ± 1.19	
**MM-6**	484 (35,700) CH_2_Cl_2_							600 (0.110)	24.37 ± 1.82	
								1000 (0.185)	27.58 ± 1.85	
								2500 (0.462)	27.82 ± 1.22	
**MM-6**	484 (35,700) CH_2_Cl_2_		DCA (10 mM)					600 (0.110)	25.42 ± 1.42	
								1000 (0.185)	27.40 ± 1.15	
								2500 (0.462)	28.95 ± 0.86	
**GZ116**	363 (58,500) 430 (27,700) 486 (23,300) DMF	TiO_2_ 12 μm (transp.) 2 μm scatt. layer	none	I^−^/I_3_^−^ TBP	PVP (Poly(N-vinyl-2-pyrrolidone)—Platinum	0.16	T5	300	16.47 ± 0.38	[50]
								600	18.01 ± 0.28	
								900	18.59 ± 0.12	
								1200	19.30 ± 0.11	
								2400	22.12 ± 0.05	
								3600	23.04 ± 0.18	
								4800	24.60 ± 0.21	
								6000	25.38 ± 1.01	
**MA1116**	346 (49,100) 409 (29,000) 472 (26,900) DMF							300	17.16 ± 0.46	
								600	18.52 ± 0.29	
								900	18.94 ± 0.36	
								1200	20.00 ± 0.53	
								2400	22.57 ± 0.36	
								3600	23.48 ± 0.37	
								4800	24.78 ± 0.48	
								6000	26.81 ± 0.16	
**GZ152**	308 (42,100) 370 (36,600) 541 (40,200) CH_2_Cl_2_	TiO_2_ 12 μm (transp.) 4 μm scatt. layer	none	I^−^/I_3_^−^ GuSCN, DMPII, TBP	platinum	-	T5	500	22.44 ± 0.41	[51]
								1000	24.08 ± 0.57	
								2000	25.12 ± 0.79	
								4000	26.45 ± 0.82	
								6000	28.31 ± 0.65	
**SBT-6**	499 (*o*-C_6_H_4_Cl_2_)	-	CDCA (10 mM)	I^−^/I_3_^−^	platinum	-	T5 fluorescent lamp	900	17.31	[50]
								1200	17.93	
								2400	21.17	
								3600	22.35	
								4800	23.30	
								6000	23.57	
**L156**	343 (49,800) 491 (25,200) CH_2_Cl_2_	TiO_2_ 4 µm (transp.) 4 µm scatt. layer	none	Cu(tmby)_2_ (TFSI)_1/2_ LiTFSI TBP	PEDOT	0.16 (mask)	Xenon light source	200	16.8 ^(c)^	[52]
								1000	21.9 ^(c)^	
								200	9.76 ^(d)^	
								1000	16.5 ^(d)^	
								200	5.17 ^(e)^	
								1000	10.6 ^(e)^	
**YK8**	479 (31,667) CH_2_Cl_2_	TiO_2_ 6 µm (transp.)	none	I^−^/I_3_^−^ HPE (GreatCell Solar)	platinum	0.1256 (mask)	T2 cool daylight fluorescent tube	700 (0.160)	22.15 ± 0.42	[53]
								1000 (0.240)	28.70 ± 1.14	
								1500 (0.360)	30.24 ± 1.23	
								2000 (0.480)	29.43 ± 0.46	
**YK9**	478 (34,454) CH_2_Cl_2_							700 (0.160)	12.49 ± 0.87	
								1000 (0.240)	14.21 ± 0.56	
								1500 (0.360)	17.84 ± 1.17	
								2000 (0.480)	20.11 ± 1.96	
**TPAA1**	364 (48,950) 415 (14,470) Toluene	TiO_2_ 12 µm (transp.) 4 µm scatt. layer	none	I^−^/I_3_^−^ HPE (GreatCell Solar)	platinum	-	warm white LED	1000	2.50 ± 0.1	[55]
		TiO_2_ 4µm (transp.) 4 µm scatt. layer		Co(bpy)_3_ (PF6)_2/3_ LiTFSI TBP	PEDOT				0.3 ± 0.06	
**TPAA8**	362 (40,300) 470 (14,470) Toluene	TiO_2_ 12 µm (transp.) 4 µm scatt. layer		I^−^/I_3_^−^ HPE (GreatCell Solar)	platinum				8.74 ± 1.3	
		TiO_2_ 4 µm (transp.) 4 µm scatt. layer		Co(bpy)_3_ (PF6)_2/3_ LiTFSI TBP	PEDOT				11.03 ± 1.7	
**LG-P1**	298 (40,400) 421 (38,500) 441 (38,200) CH_2_Cl_2_	TiO_2_ 3 µm (transp.) 3 µm scatt. layer	none	I^−^/I_3_^−^ HPE (GreatCell Solar)	platinum		daylight LED	1000	10.53	[58]
							daylight CFL	1000	6.66	
				Cu(dmp)_2_ (TFSI)_1/2_ LiTFSI, TBP	PEDOT		daylight LED	1000	0.13	[56]
**LG-P2**	325 (40,500) 424 (40,600) 444 (40,100) CH_2_Cl_2_			I^−^/I_3_^−^ HPE (GreatCell Solar)	platinum		daylight LED	1000	9.88	[58]
							daylight CFL	1000	9.19	
				Cu(dmp)_2_ (TFSI)_1/2_ LiTFSI, TBP	PEDOT		daylight LED	1000	1.41 ± 0.019	[57]
**LG-P3**	323 (43,800) 413 (42,800) CH_2_Cl_2_			I^−^/I_3_^−^ HPE (GreatCell Solar)	platinum		daylight LED	1000	9.26	[58]
							daylight CFL	1000	8.19	
				Cu(dmp)_2_ (TFSI)_1/2_ LiTFSI, TBP	PEDOT		daylight LED	1000	9.14	[56]
**LG-P4**	308 (4.33) 459 (4.08) CH_2_Cl_2_			I^−^/I_3_^−^ HPE (GreatCell Solar)	platinum		daylight LED	1000	3.68	[58]
							daylight CFL	1000	4.09	
				Cu(dmp)_2_ (TFSI)_1/2_ LiTFSI, TBP	PEDOT		daylight LED	1000	0.43 ± 0.03	[57]
**1a**	455 (26,260) CH_2_Cl_2_	TiO_2_ 13 µm scatt. layer	CDCA (03 mM)	I^−^/I_3_^−^ BMII TBP	-	0.25	OSRAM 930 lamp	1000 (0.350)	3.74	[59]
								2000 (0.708)	3.59	
**1b**	457 (22,330) CH_2_Cl_2_							1000 (0.350)	4.73	
								2000 (0.708)	4.81	
**2a**	444 (34,400) CH_2_Cl_2_							1000 (0.350)	3.93	
								2000 (0.708)	3.86	
**2b**	462 (23,550) CH_2_Cl_2_							1000 (0.350)	5.56	
								2000 (0.708)	5.56	
**3b**	434 (22,990) CH_2_Cl_2_							1000 (0.350)	3.07	
								2000 (0.708)	3.18	
**TY1**	475 (25,400) THF	TiO_2_ 10µm 4–5 µm scatt.layer	CDCA (10 mM)	I^−^/I_3_^−^ GuSCN DMPII, TBP	Platinum on carbon cloth	1	T5 fluorescent lamp	300 (0.097)	16.75 ± 0.04	[60]
								600 (0.194)	19.53 ± 0.05	
								1000 (0.324)	21.16 ± 0.05	
**CCOD-1**	334 (80,600) 519 (45,200) THF	TiO_2_ 10μm (transp) 4 μm scatt. layer	None	I^−^/I_3_^−^ DMPII TBP	Platinum	0.12	T5 fluorescent lamp	1000 (0.32)	22.1 ± 0.3	[73]
								1500 (0.48)	23.5 ± 0.4	
								2000 (0.64)	25.9 ± 0.4	
								2500 (0.80)	26.9 ± 0.3	
						1		1000 (0.32)	17.4 ± 0.2	
								1500 (0.48)	18.3 ± 0.4	
								2000 (0.64)	20.4 ± 0.2	
								2500 (0.80)	23.1 ± 0.6	
**CCOD-2**	342 (84,000) 519 (48,600) THF					0.12		1000 (0.32)	22.9 ± 0.1	
								1500 (0.48)	24.7 ± 0.2	
								2000 (0.64)	26.8 ± 0.2	
								2500 (0.80)	28.0 ± 0.2	
						1		1000 (0.32)	18.4 ± 0.4	
								1500 (0.48)	19.9 ± 0.6	
								2000 (0.64)	21.9 ± 0.2	
								2500 (0.80)	24.4 ± 0.3	

^(a)^ Light illuminance is reported in Lux; when available, the light power is reported in brackets. ^(b)^ When known, the color temperature is reported in parentheses. ^(c)^ [Cu^II^] 0.01 M; ^(d)^ [Cu^II^] 0.03 M; ^(e)^ [Cu^II^] 0.06 M.

## 3. Co-Sensitization

Along with the development of dyes with high molar attenuation coefficients and panchromatic absorption profiles, the co-sensitization of the semiconducting surface with two or more different dyes represents a promising strategy to improve the performance of the devices. The concept of co-sensitization relies on an accurate combination of dyes with complementary absorption characteristics and a suitable size relationship, to afford a broad overlap with the emission spectrum of the illumination source and an appropriate coating of the semiconducting surface. Normally, highly efficient devices employ at least two dyes, whose combination produces a synergic effect, for example, one responsible for a broad absorption over a wide spectrum yielding a high *J_SC_* but a lower *V_OC_*, and a co-sensitizer with a complementary absorption over a narrower spectrum yielding a high *V_OC_* but a lower *J_SC_*. The broader absorption spectrum and the improved shielding of TiO_2_ surface achieved in devices based on this architecture allow the enhancement of their light-harvesting ability while reducing the charge recombination processes taking place on the semiconductor; as a result, co-sensitized devices reach the best performances for DSSCs in indoor applications to date [22].

Two different techniques are usually employed for the co-sensitization of the electrode surface [37]: (1) the cocktail approach, where the co-sensitization takes place in one step by immersing the semiconductor substrate in a mixture of multiple dyes in the same solvent, and (2) the sequential approach, where the semiconducting surface is sensitized using one dye solution at the time. The cocktail approach represents the easiest fabrication technique to test new combinations of dyes but does not grant adequate control over the dye-loading ratio, possibly leading to non-optimal cell light-harvesting properties. On the other hand, the sequential approach is extremely useful for maximizing the co-sensitization efficiency of dyes that present different coordination affinities. For example, when one dye adsorbs more favorably onto the semiconducting surface than the other this can be used in the initial sensitization step, while the less coordinating dye can be used in a subsequent step to fill residual gaps on the surface. Indeed, for both methods, a complex correlation between the physical (bulkiness, molecular orientation, and shape) and chemical (polarity, nature of the anchoring group) properties of the dye and the performances of the resulting DSSCs has been shown [37,79]. Therefore, to point out which should be the preferred approach to follow in a new study is not straightforward. Most probably, a different empirical solution will have to be found in each individual case, and thus a precise optimization of the staining conditions remains mandatory to maximize cell performances.

Although hundreds of co-sensitizers have been explored as light-harvesting chromophores for outdoor performing DSSCs, to date, very few combinations of dyes have been tested under indoor or ambient light conditions. In 2017, Hagfeldt et al. reported one of the first examples of co-sensitized DSSC devices tested under indoor light illumination [80].

**Figure 29:**
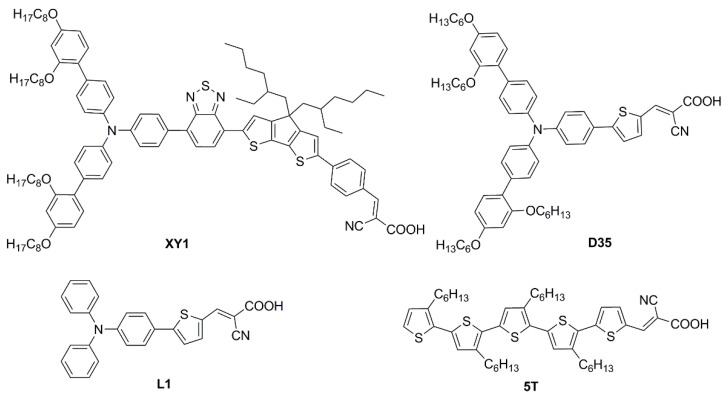
Structures of organic dyes used in DSSC co-sensitization studies [81,82,83].

**Figure 30:**
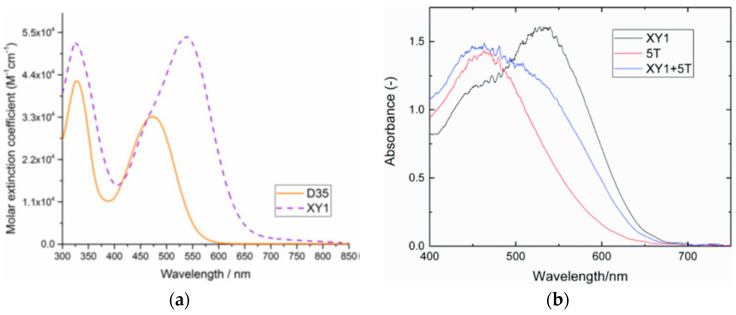
UV-Vis absorption spectra of compound **XY1** in combination with co-sensitizers (**a**) **D35** and (**b**) **5T**. Reproduced from: (**a**) Ref. [81] with permission from Springer Nature; (**b**) Ref. [81] with permission from the Royal Society of Chemistry.

The authors focused their work on **XY1** (Figure 29), a benzothiadiazole-based D-A-π-A dye with a very high molar attenuation coefficient and a spectral response extending beyond 700 nm, which was used together with the donor-π-acceptor (D-π-A) dye **D35** (Figure 29), showing a complementary absorption profile centered in the blue-green spectral region (Figure 30a). By accurately combining these two previously reported chromophores, they were able to achieve highly effective light harvesting in the visible region extending from 400 to 650 nm.

After screening the photovoltaic properties of DSSCs containing various co-sensitizer mixtures in different ratios under simulated AM 1.5 G irradiation, the best-performing co-sensitized system (4:1, **D35**:**XY1**) was then tested under ambient conditions using Osram Warm White 930 as the artificial light source. For this application, devices with a relatively large area of 2.8 cm^2^ were fabricated using Cu^II/I^(tmby)_2_ as a redox shuttle and (PEDOT)-covered FTO as the counter electrode, and investigated under two different irradiation intensities: 200 and 1000 lux. At 1000 lux, the best device achieved excellent photovoltaic metrics, such as a *V_OC_* of 797 mV, a short-circuit photocurrent of 138.0 μA cm^−2^, and an *FF* of 0.80, and produced a maximum power density of 88.5 μW cm^–2^ corresponding to a PCE of 28.9%. Such outstanding results were attributed to a number of factors, including: (i) the excellent compatibility of the spectroscopic properties of the two dyes, whose combination proved able to harvest light along the entire visible range; (ii) the more tightly packed layer formed by the two sensitizers on the semiconductor surface compared to those given by the individual dyes, limiting charge recombination phenomena; (iii) the good match of the dyes redox potentials with that of the copper-based redox mediator, causing low voltage losses in the cell and resulting in high *V_OC_*. Finally, the authors also demonstrated that their devices outperformed, in terms of efficiency and cost, other photovoltaic technologies such as GaAs thin-film solar cells under ambient light conditions, highlighting the role of DSSCs as indoor energy sources to power portable electronics.

Later, dye **XY1** was also selected as a highly spectral responsive chromophore by Freitag et al., in combination with dye **L1** (Figure 29) as the co-sensitizer [82]. From the earliest stages of the study, the photovoltaic performances of the co-sensitized DSSC, tested under simulated AM 1.5 G irradiation, largely exceeded not only those yielded by the dye alone, highlighting the synergic effect of co-sensitization but also those previously obtained by co-sensitization of **XY1** with other dyes [83]. Such a result was enabled by the perfect complementarity of the optical properties of the two compounds since yellow dye **L1** was able to absorb and convert photons also in the green-to-blue region around 400 nm, where the absorption of dye **XY1** was less intense, as demonstrated by the comparison between the IPCE spectra of the corresponding cells. Furthermore, the much smaller **L1** dye can occupy the semiconductor surface area between larger **XY1** molecules, leading to a denser monolayer that prevents the electron-back transfer from the semiconductive surface to the redox mediator, increasing the obtained photovoltage. The characteristics of photovoltaic devices under ambient light conditions were tested under illumination with an OSRAM 930 18 W fluorescent tube at 1000, 500, and 200 lux. Among all the various co-sensitization ratios investigated, the best-performing system proved to be that provided by the 1:2.5 ratio of **XY1**:**L1**. The co-sensitized device containing a Cu^(2+/1+)^(tmby)_2_-based electrolyte and (PEDOT)-covered FTO as the counter electrode showed remarkable conversion efficiencies: 34% with a power output of 103 µW cm^−2^ at 1000 lux, 32.7% with a power output of 50 µW cm^−2^ at 500 lux, and 31.4% with a power output of 19 µW cm^−2^ at 200 lux. In addition, the study demonstrated that by employing a small array of **XY1**:**L1**-sensitized cells, it was possible to provide sufficient power from ambient light to feed IoT devices capable of sensing and communicating data within a wireless network. As a striking example, it was shown that a 5-cell array (16.0 cm^2^ total) could be used to power a wireless node equipped with a sensor for indoor temperature measurement during 12 days of a simulated day–night cycle, consisting of 16 h of 1000 lux illumination and 8 h of darkness, using the energy stored in an AVX 6.0 V 0.47 F supercapacitor to ensure data transmission during “night” periods.

As can be seen from the reported examples, dye **XY1** represents a valid choice in terms of high-responsive chromophores for indoor applications. The main issue related to its employment is the costly synthesis, estimated by Robertson et al. [81]. at 868 USD/mmol (537 USD/g), higher than the commercial price of the classic organometallic dye **N719**. In this context, the co-sensitization technique is proposed by the authors as a simple and effective method to reduce the associated fabrication costs of DSSCs, which is mostly related to dye synthesis. The simple and less-expensive π-A dye **5T** (Figure 29) presented a complementary UV-Vis absorption spectrum to that of **XY1** (Figure 30b), along with a more positive ground-state oxidation potential than the redox potential of the Cu^(2+/1+)^(tmby)_2_ complex (1.08 V vs. 0.87 V), making it a suitable co-sensitizer for indoor applications. Preliminary photophysical measurements under simulated illumination conditions of 1 Sun revealed the complementary effect of **XY1** and **5T** in the 1:1 co-sensitized devices, reaching photoelectric conversion efficiencies similar to the DSSCs using only **XY1,** or even superior when tested under 0.1 Sun. As a result of this trend, **XY1+5T**-co-sensitized devices with a larger active area of 3.2 cm^2^ were fabricated and tested with OSRAM 930 18 W fluorescent tube at 1000 lux and reached a remarkable PCE of 29.2%. Furthermore, from the cost estimation of each dye and the estimated molar equivalent of **XY1**, **5T**, and **XY1+5T** on TiO_2_ films, it was demonstrated that the cost of dye per unit area can be reduced to ca. 70% by blending **XY1** and **5T**. This result was mainly due to the amount of **5T**, ca. 1.7 times higher than that of **XY1** dye incorporated in the **XY1+5T** film, which led to an overall cheaper device in comparison to the one containing only the **XY1** dye.

**Figure 31:**
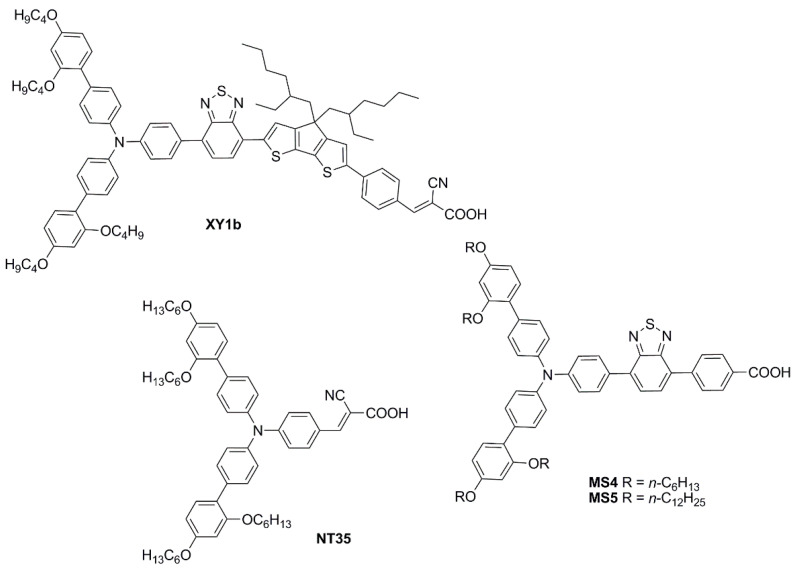
Structure of co-sensitizers **MS4** and **MS5,** of reference dye **NT35** and broadly-responsive dye **XY1b** [22].

Recently, the development of co-sensitized DSSCs with high *V_OC_* has been the focus of increasing attention by the scientific community, as can be seen in the study conducted by Grätzel et al., where two new donor-acceptor dyes, named **MS4** and **MS5** (Figure 31), capable of giving cells with high photovoltages, have been proposed [22].

Both molecules were successfully synthesized using mainly Suzuki–Miyaura and Buchwald–Hartwig cross-coupling reactions, and completely characterized in solution. As shown in Figure 31, the designed dye **MS4** carries the same donor group of reference dye **NT35** but presents a 4-(benzo[*c*][1,2,5]thiadiazol-4-yl)benzoic acid (BTBA) instead of a cyanoacrylic acid as the acceptor group. Such a structure allows a higher dye loading on the semiconductor, potentially depressing interfacial charge recombination thanks to better surface coverage. As a result, a higher photovoltage (1.17 V) was observed when testing DSSCs built with this compound under simulated AM 1.5 irradiation in combination with Cu^(2+/1+)^(tmby)_2_ as a redox mediator. By further elongating the end chains of the donor of **MS4**, the resulting **MS5** showed an enhanced *V_OC_* of 1.24 V. Benefiting from its higher photovoltage and the strong capacity of retarding interfacial charge recombination, **MS5** was selected as the most promising dye for co-sensitization. Co-sensitized DSSCs with an active area of 2.8 cm^2^ were decorated by complementary dyes **XY1b** and **MS5** as sensitizers and tested with Cu^(2+/1+)^(tmby)_2_ as the electrolyte and (PEDOT)-covered FTO as the counter electrode. Remarkably, they achieved notable photovoltaic metrics when irradiated with Osram 930 Warm White light under variable illuminance intensities (200, 500, and 1000 lux). The best-performing devices reached a PCE of 34.5% with a maximal power output of 307.4 µW cm^−2^ and an impressive *V_OC_* of 0.98 V. The authors carried out an in-depth analysis of the results obtained with the co-sensitized cells and concluded that their outstanding performances could ultimately be led back to the formation of a compact and ordered monolayer on the TiO_2_ surface, enabled by the complementary structures of **X1b** and **MS5** and the steric protection provided by their aptly placed alkyl substituents. This resulted in a series of positive effects, including: (a) a higher dye loading compared to the case of **X1b** alone, (b) a reduced rate of charge recombination and a longer lifetime of injected charges, (c) a higher dye regeneration efficiency, and (d) a better ideality factor, all contributing to the increased cell efficiency. The obtained result thus showed once again the importance of judicious molecular engineering of the dyes to improve the performances of the devices.

**Figure 32:**
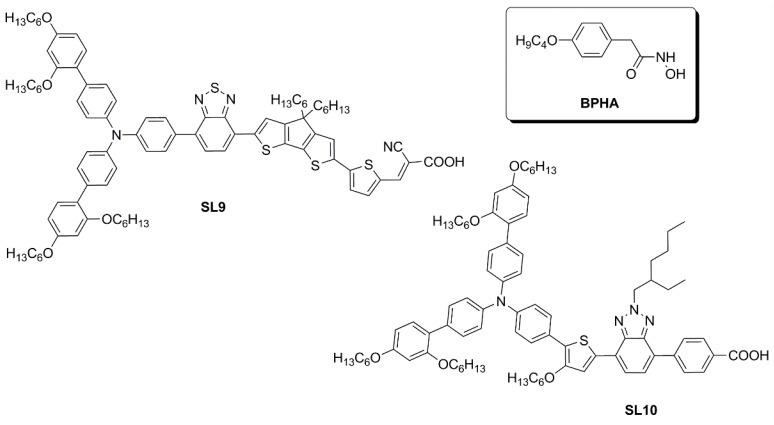
Structure of high-responsive chromophores **SL9** and co-sensitizer **SL10**.

Besides the complementary optical absorption properties and a suitable size ratio between the two sensitizers, good control over the adsorption and self-assembly processes, as well as appropriate properties of the formed monolayer are also crucial for enhancing DSSCs performances, as proven by Grätzel et al. in their most recent report where two new chromophores were proposed: dyes **SL9** and **SL10** (Figure 32) [84].

**Figure 33:**
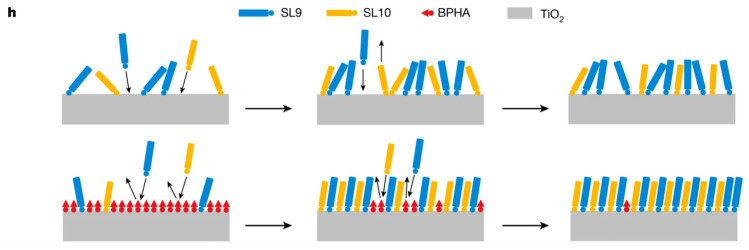
Schematic mechanism of dye adsorption and co-sensitization with **SL9**/**SL10** mixtures, and effect of BHPA on the adsorption geometry. Reproduced from Ref. [84] with permission from Springer Nature.

Dye **SL9** was designed as a high-responsive dye and exhibited a broad absorption spectrum across the entire visible domain except for a spectral window around 400 nm. To guarantee full coverage of the visible region, dye **SL10** was designed and synthesized as a co-sensitizer. Along with the desired complementary absorption proprieties, co-sensitizer **SL10** presented a bulky triarylamine donor group, designed to hinder interfacial charge recombination. Besides the accurate molecular engineering of the dyes, the authors demonstrated how the pre-adsorption of 2-(4-butoxyphenyl)-*N*-hydroxyacetamide (BPHA) on the TiO_2_ surface before co-sensitization can retard the uptake of dye molecules while at the same time inhibiting the desorption of the co-sensitizers, generating a dense and ordered molecular packing (Figure 33). As a result, the pre-treated co-sensitized DSSCs showed enhanced photovoltaic performances compared to the not pre-treated ones. When illuminated by a 4000 K LED lamp at 1479 lux light intensity, the BPHA-pretreated DSC, with an active area of 2.8 cm^2^, achieved a *Voc* of 1.04 V, a *Jsc* of 155.3 µA cm^−2^, and an FF of 80.4% yielding a power density of 129.6 µW cm^−2^ that corresponds to a PCE of 30.1%.

In conclusion, the co-sensitization approach for the photoanode staining of dye-sensitized solar cells (DSSCs) has demonstrated significant potential for enhancing device performances, particularly in indoor lighting conditions. This is especially due to the possibility of maximizing artificial light harvesting by achieving an overall light absorption profile as broad as possible. Thus, by combining dyes with complementary absorption properties, DSSCs have achieved impressive photovoltaic metrics reaching the current record efficiencies. As shown, dyes must be carefully selected to have productive matching, and the staining technique is also a crucial aspect for achieving good coverage of the semiconductor surface. In this context, the use of advanced sensitization procedures, able to yield a more compact, ordered, and dense dye monolayer on the semiconductor surface, as recently shown by Grätzel et al. [84], appears particularly promising to improve device performances even further. Finally, special attention should be dedicated to reducing the cost of materials and fabrication processes, for example, by employing low-cost co-sensitizers [81] or changing the electrode compositions, which can make DSSCs more accessible for wider applications.

Materials used, essential experimental details, and photovoltaic properties of the devices prepared in the co-sensitization experiments discussed in this chapter are reported in Table 4.

## 4. Concerted Companion Dyes

**Figure 34:**
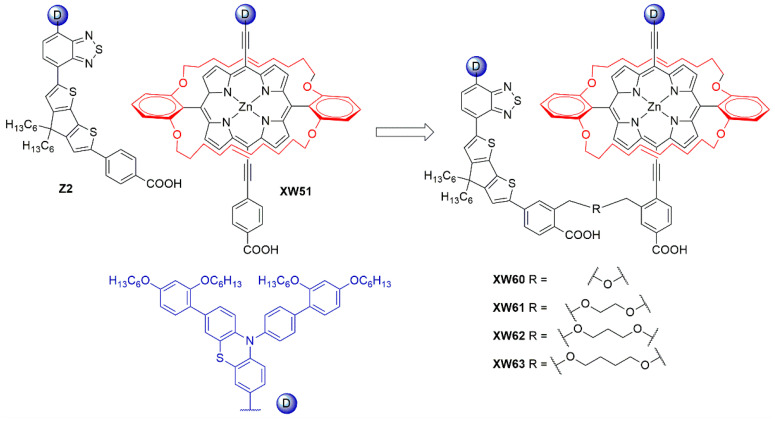
Molecular structures of dyes **Z2**, **XW51**, and CC dyes **XW60**–**XW63** [88].

Despite the success achieved by the co-sensitization strategy [37], such an approach still presents some substantial drawbacks, especially related to the need to optimize several practical parameters such as the dye loading procedure (i.e., cocktail or sequential), the adsorption sequence [85], the staining solvents, as well as the concentration, stoichiometric ratio and adsorption time of the dyes, with the aim to obtain an optimal distribution of the adsorbed sensitizers on the TiO_2_ film [79]. To address these issues, Xie and coworkers recently proposed an innovative approach, based on the employment of a novel class of sensitizers, named “concerted companion dyes” (abbreviated as “CC dyes”). They feature two covalently connected but independent compounds, both capable of anchoring on the semiconductor, thus realizing a precise “intramolecular” co-sensitization process, an alternative to the commonly used “intermolecular” one [86]. Thanks to their structure, this kind of sensitizer might represent a significant step forward for the development of ambient light DSSCs, since they are designed to ensure panchromatic absorption, which is essential for maximizing indoor light harvesting. Furthermore, they are expected to improve not only the performances of the cells but also their stability, which is a crucial point in view of practical applications. Such an approach was first put into practice by linking the doubly wrapped porphyrin dye **XW51** [87], featuring a donor–π–acceptor (D–π–A) configuration, with the organic dye **Z2** through flexible chains of various lengths, to give the four new dyes **XW60-63** (Figure 34) [88].

Dye **Z2** was designed to fill up the absorption defects of bulky porphyrin dye **XW51** and exhibited two broad absorption bands in the 350−440 and 470−640 nm regions. As a consequence, the final CC dyes **XW60**−**XW63**, besides featuring typical Soret and Q bands of the porphyrin component, also presented medium absorption bands around 400 and 500 nm, exhibiting a panchromatic light-harvesting ability. All DSSCs prepared using dyes **XW61**−**XW63** exhibited comparable high efficiencies of 11.6−11.7% under 1 Sun irradiation, using I^−^/I_3_^−^ as the redox shuttle, demonstrating the relative insensitivity of the system toward the bridge length. Nevertheless, **XW60**, having just a single oxygen atom as the bridging group, provided a comparably lower PCE of 8.8%, which was ascribed to the steric clash between the two individual units, preventing their correct absorption on the semiconductor surface and causing excessive charge recombination. Remarkably, the coadsorption of **XW61** with CDCA afforded an improved efficiency of 12.4% thanks to a more compact coverage of the surface hindering charge recombination. Furthermore, by using a cobalt-based electrolyte, an efficiency of 10.7% was achieved with the same dye, higher than those of around 7.4−9.6% obtained for individual dyes of **XW51** and **Z2** as well as the co-sensitized **XW51** + **Z2** systems. Finally, the strong adsorption through two anchoring carboxylic groups seems also to be favorable for enhancing the durability of the solar cells.

**Figure 35:**
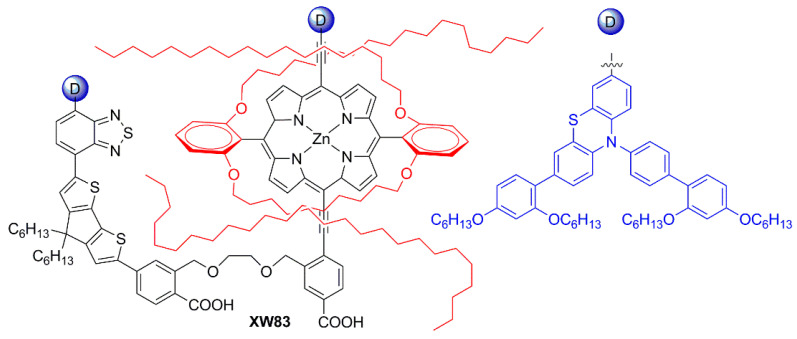
Molecular structure of dye **XW83** [91].

Subsequent works from the same group reported that extended panchromatic absorption and excellent light harvesting can be achieved by introducing strong donor moieties, such as the fluorenyl indoline moiety, and bulky substituents on the porphyrin [89] or by optimization of the organic dye structure [90]. Moreover, it has been shown that wrapping the porphyrin macrocycle with alkoxy chains at the ortho-positions of the phenyl groups can lead to the obtainment of high photovoltaic performances without using any co-adsorbent. For instance, compound **XW83**, decorated with C18 chains (Figure 35), exhibited high *V_OC_* (784 mV) and PCE (12.2%) under 1 Sun in the presence of an iodine-based electrolyte [91]. Therefore, the efficiency provided by **XW83** alone was comparable to that obtained with **XW61** in combination with CDCA, thus helping to simplify the cell fabrication procedure. According to the authors, that was due both to the anti-charge recombination and anti-aggregation effect of the alkoxy wrapping chains, which was demonstrated by means of electrochemical impedance spectroscopy (EIS) experiments.

**Figure 36:**
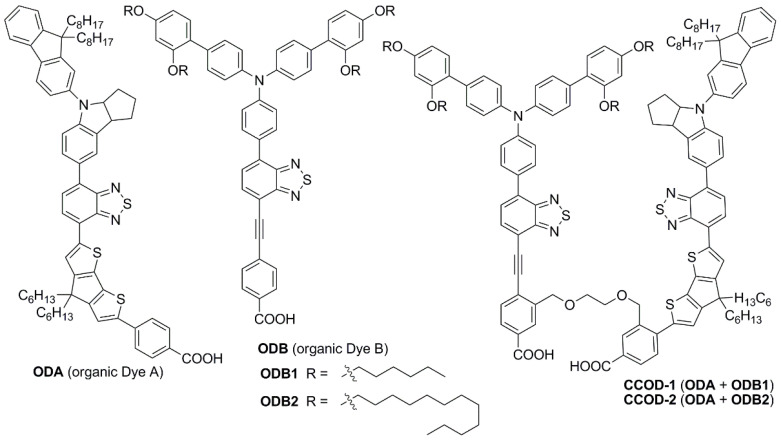
Molecular structures of dyes **ODA**, **ODB1**, **ODB2,** and CC dyes **CCOD-1** and **CCOD-2** [88].

**Figure 37:**
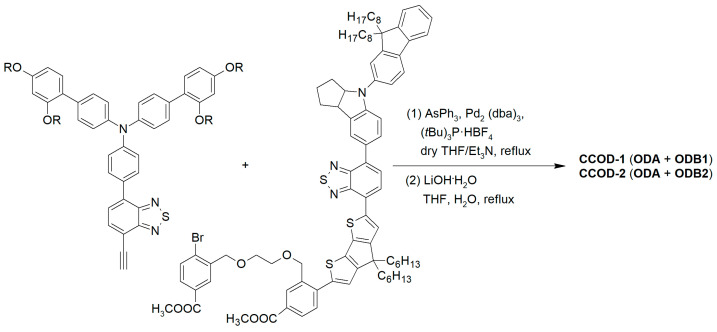
Synthetic route for dyes **CCOD-1** and **CCOD-2** [88].

Such an approach was demonstrated to be very promising for fabricating DSSCs able to efficiently harvest the energy of indoor lamps. However, for this kind of application, it was inferred that porphyrin dyes, suffering from a severe aggregation which is usually responsible for large voltage loss and low *V_OC_*, were probably unfavorable for harvesting the energy of dim light. Accordingly, a new class of CC dyes was designed, based on two organic units covalently linked together. In particular, the dyes **ODA**, **ODB-1,** and **ODB-2,** bearing fluorenyl-indoline and triarylamine-based donors, respectively, were synthesized and characterized (Figure 36). Dyes **CCOD-1** and **CCOD-2** were obtained from suitable precursors via Sonogashira coupling, followed by hydrolysis reactions (Figure 37) [88].

The two dyes, assembled with the two spectrally complementary units, exhibited intense light absorption from 300 to 650 nm, which was even broadened after adsorption on TiO_2_, well covering the emission of an indoor T5 fluorescent lamp. The photovoltaic performances were initially evaluated under simulated sunlight (AM 1.5 G), using **N719** as the reference dye, and satisfactory PCE values were found, higher than those provided by the isolated dyes **ODA**, **ODB1**, and **ODB2**. In particular, **CCOD-2**-sensitized DSSCs afforded a PCE of 10.4%, which is higher than those of devices built with **CCOD-1** (9.82%) and reference dye **N719** (9.81%). However, the best photovoltaic performance was obtained under the 1000–2500 lux illumination of a fluorescent lamp. Photovoltaic parameters of DSSCs built using the simple organic dyes **ODA**, **ODB-1**, and **ODB-2**, were compared with those of cells containing the previously reported CC dyes **XW61** (Figure 34) and **XW70-C8**, and the reference dye **N719**, under the same conditions. All the devices showed optimal PCEs under 2500 lux illumination: in particular, a boosted PCE of 28.0% was achieved for **CCOD-2** under the T5 light source, which was slightly higher than that obtained with **CCOD-1** due to a reduced charge recombination allowed by the longer alkyl chains on the donor. Furthermore, such value was considerably higher than those obtained with the porphyrin-based CC dyes (up to 22.4%), due to the lower *V_OC_* and the worse match with the light source emission of the latter, as mentioned above. Therefore, the PCE provided by **CCOD-2** was comparable with the highest efficiencies reported at the time for organic dyes under indoor conditions [22,60,64,80,92]. Using dye **CCOD-2**, the corresponding devices with larger active areas (1 cm^2^) were also fabricated, affording a PCE of 24.4%. Aging tests of the devices were carried out by continuous irradiation under simulated sunlight at 25 °C for 500 h. Under these conditions, **CCOD-2** showed the best stability, retaining 92% of the initial PCE, in contrast with what was found with the mono-anchored dyes, which revealed efficiency losses in the 14–21% range [88]. The latter results appear particularly promising since they demonstrated that CC dyes are, at least in principle, employable in larger-scale photovoltaic devices and have the potential to yield DSSCs with improved durability, which are both relevant issues in view of the practical application of this PV technology.

In summary, CC dyes represent a new straightforward approach to achieving highly performing DSSCs for indoor applications, not needing any anti-aggregating agent or co-adsorbent, thus allowing an easy cell fabrication procedure, a simpler optimization of the staining parameters compared to the co-sensitization strategy, and possibly yielding excellent photostability. Currently, the main issue related to their use appears to be the need to synthesize relatively complex molecular dyads, obtained by covalently linking two dyes whose preparation usually calls for a multistep synthetic procedure per se. Since this could likely limit the access to large dye libraries, in this context a more focused approach appears preferable, in which only selected sensitizers are combined based on the preliminary assessment of their individual properties, as already successfully demonstrated by the examples reported above.

Materials used, essential experimental details, and photovoltaic properties of the devices prepared with the dyes discussed in this chapter are reported in Table 3.

## 5. Characterization of Photovoltaic Modules for Low-Power Indoor Application

**Figure 38:**
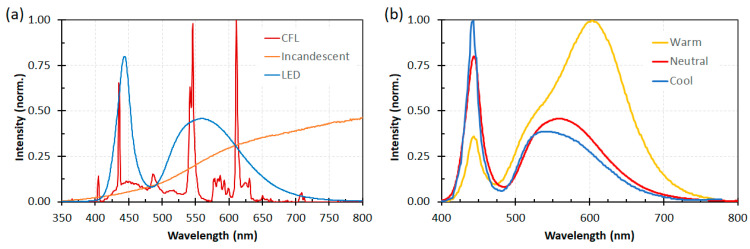
(**a**) Typical spectra of the most used indoor light sources (LED, Incandescent, CFL). (**b**) Warmer LEDs showing a red-shift and a lower blue peak compared to cooler LEDs.

In addition to the challenges represented by the design, synthesis, and application of optimized molecular sensitizers, as shown in the previous sections, a relevant issue in the area of indoor photovoltaics (IPV) is still represented by the identification of correct and comparable procedures for the assessment of device efficiencies. Indeed, standardized characterization of IPV devices in indoor conditions remains challenging due to a lack of an industry-standard spectrum for incident light, making it difficult to compare results across the literature [49,93]. Moreover, photovoltaic devices under 1 Sun illumination have additional standards for environmental parameters such as humidity, temperature, and irradiance, outlined in the ISOS protocols [94]. The latter cannot be applied to IPV as they always operate at room temperature, with varying humidity levels, and with different light spectra. Although more and more indoor environments are illuminated with white LEDs, traditional light sources such as incandescent, compact fluorescent (CFL), and halogen lamps (Figure 38) should also be considered when reporting about the efficiency of IPVs.

The most important parameter describing the performance of a solar cell is its efficiency. It is obtained by registering a current/voltage (*J*/*V*) curve under illumination, sweeping a voltage from 0 V, where the current is at short circuit (*Jsc*) until the current reaches zero and the device is considered at open-circuit (*Voc*). At some point in between, the cell generates its maximum power (MPP) at *Vmax* and *Jmax*, which can be used to calculate the power conversion efficiency (PCE) as shown in Equation (1):

(1) $$PCE=\frac{V_{max}{\;J}_{max}\;FF}{P_{in}}$$

where *P_in_* is the incident irradiant power and *FF* (Fill Factor) is defined as:

(2) $$FF=\frac{V_{oc}\;J_{sc}}{V_{max}\;J_{max}}$$

For devices under sunlight, *P_in_* is set to 1000 W/m^2^ as per the AM1.5 G standard solar spectrum. However, IPVs are illuminated by a mix of sunlight and various artificial lighting during the day and only artificial light during the night.

The operating point of IPVs varies greatly throughout the day as the intensity of indoor light sources is roughly 3 orders of magnitude lower than direct sunlight [38]. Except for incandescent bulbs, the emission spectrum is concentrated in the visible region (400–700 nm). In contrast to sunlight, all of the collected light falls within the quantum efficiency (*EQE*) curve of typical DSSCs. The match between the emission spectrum of the light source and the *EQE* of the device can result in efficiencies of well over 25% for typical DSSCs [22]. In order to perform accurate and repeatable measurements, standards have been established such as IEC 60904-3:2019 [95] for measurements under outdoor lighting. Measurements for indoor environments lack such a standard and it is, therefore, difficult to compare measurement results with the literature.

### 5.1. Illuminance vs. Irradiance

The Sun spectrum ranges from wavelengths in UV (<400 nm) to far in the infrared region (>800 nm). The intensity is always given in irradiance with units W/m^2^, with direct sunlight (defined in the AM 1.5 G spectrum) having 1000 W/m^2^. A significant portion of this spectrum resides within the infrared region that cannot be absorbed by typical photovoltaic devices. Equally noteworthy, especially for indoor perspectives, is the fact that this infrared segment also lies beyond the range of human visual perception. For this reason, in the indoor lighting context, we rather consider *Illuminance* with units of lux (lumens per square meter), to quantify the light intensity. Illuminance is defined as:

(3) $$Illuminance=k\int_{390\;nm}^{830\;nm}P\left(\lambda \right)\;E\left(\lambda \right)\;d\lambda$$

**Figure 39:**
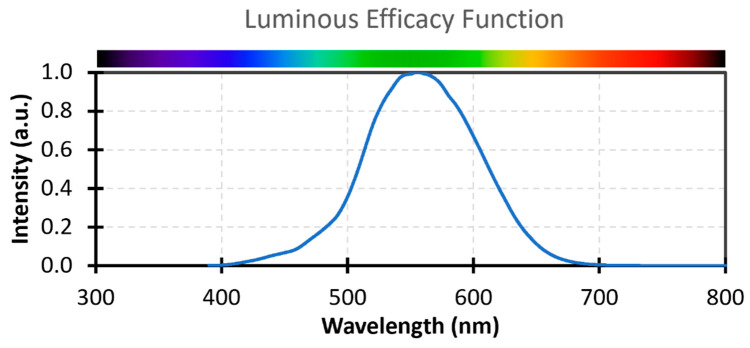
Photopic luminosity function used to convert irradiance to illuminance [96].

With k defined as 683 lumens/Watt, $P\left(\lambda \right)$ radiance of the light source and $E\left(\lambda \right)$ the photopic luminosity function (Figure 39) [96].

From the definition (Equation (3)), any change in illuminance can be directly observed by humans. Although illuminance can be a useful indication of the intensity of the light, it must be remembered that it is not possible to convert illuminance to irradiance without knowledge of spectral shape, and thus this parameter is not useful to calculate the efficiency of photovoltaic devices; to this end, irradiance should always be provided instead [97].

### 5.2. External Quantum Efficiency

To understand how efficiency is affected by the spectrum of light, the quantum efficiency of the devices should be measured. In this test, the device is illuminated with monochromatic light of known intensity at each wavelength of interest and the ratio of generated electrons to incident photons is measured. The generation of a monochromatic light source is often carried out by placing a monochromator between the device and a broad spectrum lamp that covers at least the band gap of the device. Xenon arc lamps are preferred due to their high intensity, broad spectrum, and similarity to the AM1.5 G solar spectrum.

The generated current is then measured and compared to the known number of photons of the incident light.

(4) $$EQE\left(\lambda \right)=\frac{current(\lambda )/charge\;of\;one\;electron}{power(\lambda )/energy\;of\;one\;photon}=\frac{I\left(\lambda \right)/e}{P\left(\lambda \right)/h\nu }$$

With $I\left(\lambda \right)$ the current generated by the device, and $P\left(\lambda \right)$ the irradiance of the incident light.

The *EQE* is limited mainly by the bandgap of the active material as the sharp drop-off, reflections by the other layers, and recombination of generated charges either at the surface or interlayers. While for outdoor devices an *EQE* as wide as possible is desirable, indoor applications remain restricted in the visible region and therefore high efficiencies are only required within this range.

**Figure 40:**
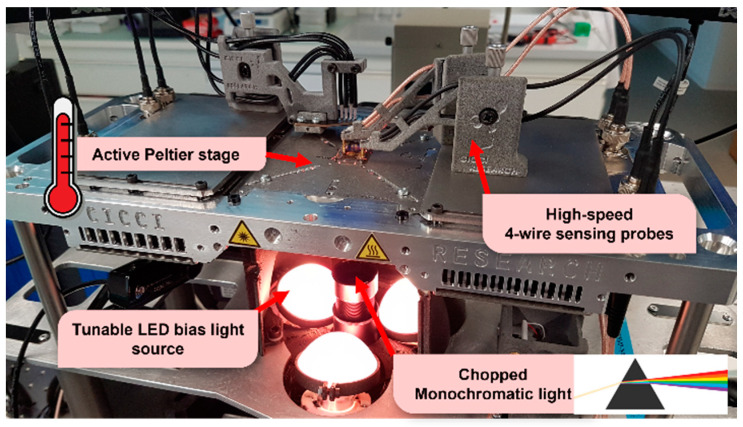
Example of a hardware setup for an *EQE* measurement with LEDs for a bias light, probes for current measurement, and a thermal stage for temperature control.

Figure 40 shows an example of a hardware setup for an *EQE* measurement. A device is placed in the middle of a measuring stage with the active side facing down. Monochromatic light is generated via an external monochromator setup and directed from the bottom of the stage to the device through an aperture. Masks can be added between the aperture and the device for shadowing. The generated current is measured with probes connected to a source meter unit (SMU) keeping the device at short-circuit current. The intensity of the source lamp is often not consistent over time due to the aging of the lamp. Calibration of the lamp using a reference photodiode with a known responsivity, units A/W, at each wavelength is performed to ensure accurate measurements.

As the *J_SC_* of the device is measured at each wavelength during an *EQE* test, the total *J_SC_* under illumination can be obtained from the *EQE* measurement:

(5) $$J_{sc}=q\int P\left(\lambda \right)\cdot EQE\left(\lambda \right)\cdot \frac{\lambda }{hc}d\lambda$$

Although this should match the *J_SC_* obtained from a *J*/*V* scan, this is often not the case [98]. One reason may be the different behavior of generating charges at different light intensities between an *EQE* and *J*/*V* measurement, especially when the photocurrent does not scale linearly with light intensity [99]. A bias light can be added during an *EQE* measurement to replicate the measurement conditions during a *J*/*V*. Since the relatively weak monochromatic light is superimposed on the bias light, the small perturbation results in an approximately linear behavior of the photocurrent. Figure 40 shows a tunable LED-based bias light source using diffusing lens caps to provide homogeneous light to the device. This light can be tuned both in intensity and spectrum to get accurate *EQE* values under the proposed working conditions.

An *EQE* measurement with monochromatic light does not necessarily simulate an accurate real-world operating condition where the device is continuously illuminated. The introduction of a bias light during the measurement increases the total density of photogenerated carriers within the device. Especially in devices with suboptimal built-in electric fields or poor carrier transport properties, this increased carrier density can significantly improve the collection of photogenerated carriers. Such improvement occurs thanks to the bias light which helps to fill the trap states, which are imperfections in the photovoltaic material that can capture and immobilize charge carriers, hindering their movement and reducing the efficiency of the device [100].

When a bias light is used, however, the monochromatic light source is relatively weak, making it difficult to distinguish the photocurrent actually generated by the incident light. While the *EQE* can still be measured, since it is compared to a reference photodiode, adding a bias light results in a lower signal-to-noise ratio. To resolve this issue, a chopper, and a lock-in amplifier can be introduced into the system to separate the signal from the noise. However, for devices with slow response times like DSSCs, the chopping frequency can affect the measured current, and in turn the *EQE*, if the period is longer than the settling time of the photocurrent [101].

### 5.3. Future Developments

The lack of standard test conditions (STC) makes it difficult to observe a trend in the performance of IPVs in the literature and set a precise performance target. To address these challenges, it would be essential to establish international standards for indoor PV characterization. This would involve defining a set of standard test conditions tailored for indoor lighting environments (e.g., specific lux levels representative of typical indoor lighting scenarios), developing efficiency metrics reflecting performance under these conditions, and establishing stability and durability tests under indoor environments. As a general suggestion for future characterizations, we therefore propose the use of at least a neutral LED as a light source (Figure 38b), at various fixed intensities, e.g., 200, 600, and 1000 lux. As the efficiency of a device is greatly affected by the spectral shape of the light source, both it and the *EQE* should be provided to accurately compare efficiency values across the literature. For completeness, a standard configuration should also involve a homogeneously lighted surface and a controlled environment in which samples are kept at room temperature and a humidity of about 40% [102]. Having such a standard testing condition will more clearly highlight the research and development trends of IPVs and can help speed up the development of future devices. These protocols can then evolve into standards for the production of commercial IPVs ensuring consistent product quality.

## 6. Conclusions

In conclusion, in this review, we have recapitulated the most relevant results recently reported in the field of new organic photosensitizers for application to indoor DSSCs. The works cited here testify that this is currently a very lively research subject, and substantial advancements have been achieved in the last few years.

Despite that, it must be recognized that the investigation of new structural motifs for indoor DSSC dyes has been somewhat fragmented, and has mostly been carried out by a few research groups focusing on specific compound classes, among which the most represented ones have been fluorene, anthracene, and fused pyrazine derivatives. As a consequence, it is currently difficult to draw a clear evolution trend for the dyes used in this kind of application or identify some “privileged” structures that the upcoming research should focus on. Clearly, to make further progress, it will be necessary to explore a wider portion of the chemical space and to perform broader, across-the-board comparisons between structurally diverse dye classes.

Nevertheless, as pointed out in Section 2.5, it is still possible to outline some general considerations and potential guidelines to help the development of better-performing sensitizers. First of all, from the literature, it clearly emerges that although design criteria for indoor DSSC dyes are based on the same general principles governing the development of sensitizers for outdoor applications, a specific fine-tuning of their structural features is required to maximize performances under artificial and weak illumination. In this regard, precise matching of the dye absorption spectra with the emission profile of the chosen light source is crucial to ensure efficient light harvesting, which is especially relevant under low light conditions. Furthermore, due to the comparatively low density of charge carriers injected in the semiconductor compared to outdoor conditions, it becomes essential to introduce onto the dye structures a sufficient number of bulky substituents, such as long alkyl chains, able to shield the surface from the electrolyte, thereby depressing charge recombination phenomena. Finally, considering that the highest theoretical efficiencies for indoor DSSCs can be reached for relatively large band-gap values approaching 2.0 eV [23], it appears promising to develop dyes with comparatively high ground-state oxidation potentials and use them in combination with copper-based redox mediators since the latter has been demonstrated to yield substantially higher *V_OC_* values compared to traditional, iodine-based, electrolytes.

As an alternative to the use of single organic sensitizers, different approaches have been tested to maximize light harvesting and improve coverage of the semiconductor surface. The most traditional of them is the co-sensitization strategy (Section 3), which involves the concomitant adsorption of two (or more) sensitizers on the same photoelectrode. By judiciously choosing compounds with compatible spectroscopic and structural properties, excellent results have been achieved, with cell efficiencies surpassing the 30% mark. In particular, the use of dye **XY1** has been very successful, since it has demonstrated compatibility with a wide range of co-sensitizers as well as copper-based redox shuttles, leading to *V_OC_* values close to 1.0 V. On the other hand, a significant drawback of the co-sensitization strategy is represented by the necessary tedious optimization of the staining procedure, involving the screening of several different parameters such as dye concentrations and stoichiometric ratio, staining solvent, adsorption times, etc., which could slow down further progress.

To overcome this issue, the approach based on the use of the so-called “concerted companion dyes” has been recently proposed, as shown in Section 4. Indeed, the concept of forming a covalent connection between two dyes with complementary optical and structural features appears very promising, as it ensures a broad coverage of the visible light spectrum as well as an optimal adsorption ratio of 1:1 in each staining condition. Nevertheless, this approach finds a limitation in the comparatively more complicated synthetic procedures required to access the dyes, which should be taken into account when designing new structures.

Besides the challenges associated with the design, synthesis, and application of the sensitizers, a relevant issue in the area of indoor photovoltaics (IPV) is still constituted by the identification of correct and comparable procedures for the measurement of device efficiencies. For this reason, in the last section of this review, the problem of measurement standardization has been briefly introduced, and the most important parameters used to define the cell performances and the light source properties have been described. The section has then been concluded by providing some suggestions regarding the proper characterization methods of indoor PV systems and highlighting their differences compared to (simulated) outdoor conditions.

At the moment, indoor PV probably represents the most promising field of application of DSSCs, where they have been proven to outperform other, more well-established photovoltaic technologies. This is even more relevant when considering that the indoor PV sector is expected to undergo significant growth in the next few years, as a result of the wide diffusion of IoT devices and systems in various environments, such as homes, offices, and factories. To meet this challenge, further research efforts will be required to bring DSSC performances to the market level. In this regard, as a final consideration, it must be remarked that future commercialization of this technology will only be possible if research, besides efficiency, will also concentrate on improving long-term dyes and cell stability, as well as developing suitable and sustainable processes for its scale-up from test cells to modules and panels.

## Figures and Tables

**Table 1:** *V_OC_* values (in volts) registered for DSSCs built with dyes **TKU-1-6** under different illumination conditions [50].

	TKU-1	TKU-3	TKU-6	TKU-2	TKU-4	TKU-5
LED	0.50	0.53	0.56	0.57	0.58	0.59
T5	0.49	0.55	0.55	0.55	0.60	0.60
AM 1.5 G	0.63	0.69	0.60	0.59	0.65	0.63

**Table 2:** Efficiency of three **RY**-series dyes under 2500 lux irradiation by different indoor lighting sources [52].

Light Source	PCE (%)		
	RY1	RY3	RY5
CWF	16.06 (±0.77)	17.49 (±0.79)	18.29 (±0.70)
TL84	17.94 (±0.91)	20.83 (±0.84)	19.74 (±0.76)

**Table 4:** Materials used and photovoltaic properties of the devices prepared in the co-sensitization experiments discussed in this review (Section 3).

Dye 1 Solvent *λ_abs_* (nm) *ε* (M^−1^/cm^−1^)	Dye 2 Solvent *λ_abs_* (nm) *ε* (M^−1^/cm^−1^)	Staining Procedure	Semi Conductor Layer	Additive	Electrolyte	Counter Electrode	Active Area (cm^2^)	Lamp Type ^(a)^	Illumin-ance (Lux) ^(b)^	PCE (%)	Ref.
**N719** BuOH/MeCN 0.3 mM 535 (16,300)	**RY3** CH_2_Cl_2_ 0.3 mM 450 (45,000)	Sequential **N719** (8 h) **RY3** (4 h)	TiO_2_ 12 μm (transp.) 6 μm scatt. layer	none	I^−^/I_3_^−^ TBP	platinum	0.28 (mask)	TL84 (4100 K) CWF (4150 K)		24.45 ± 1.12 26.19 ± 0.85 21.67 ± 0.81 25.19 ± 0.63	[38]
									1000		
									2500		

**N719** BuOH/MeCN 0.3 mM 535 (16,300)	**YS1** CH_2_Cl_2_ 0.3 mM 384 (48,400)	Sequential **N719** (6 h) **YS1** (6 h)	TiO_2_ 12 μm (transp.) 6 μm scatt. layer	none	I^−^/I_3_^−^ TBP	platinum	0.28 (mask)	TL84 (4100 K) CWF (4150 K)	2500	20.82 ± 0.49 19.50 ± 0.55	[39]
	**YS2** CH_2_C_l2_ 0.3 mM 417 (34,800)	Sequential **N719** (8 h) **YS2** (4 h)						TL84 (4100 K) CWF (4150 K)		23.76 ± 0.96 21.56 ± 0.89	
**MM-6** CH_2_Cl_2_ 0.3 mM 484 (35,700)	**MM-3** CH_2_Cl_2_ 0.3 mM 427 (80,600)	Sequential **MM6** (8 h) **MM3** (4 h)	TiO_2_ 12μm (transp.) 6 μm scatt. layer	none	I^−^/I_3_^−^ TBP	platinum	0.28	TL84	600 (0.110)	27.76 ± 1.39	[49]
									1000 (0.185)	28.74 ± 1.06	
									2500 (0.462)	30.45 ± 1.06	
**D35** 0.08 mM MeCN/ *t*BuOH 1:1 445 (70,100)	**XY1** 0.08 mM MeCN/ *t*BuOH 1:1 552 (56,500)	Cocktail **D35**:**XY1** 4:1 for 16 h	TiO_2_ 4 μm (transp.) 4 μm scatt. layer	none	Cu(tmby)_2_ (TFSI)_1/2_ LiTFSI, TBP MeCN	PEDOT	0.1582	Osram Warm White 930	200	25.5	[61]
									1000	28.9	
					Cu(tmby)_2_ (TFSI)_1/2_ LiTFSI, TBP MeCN				200	22.3	
									1000	27.4	
**T5** 0.1 mM CDCA 0.4 mM CHCl_3_/EtOH 3:7 478 (39,000)	**XY1** 0.1 mM CDCA 1mM CHCl_3_/EtOH 3:7 552 (56,500)	Cocktail: **T5**:**XY1** 1:1 for 16 h	TiO_2_ 4 μm (transp.) 4 μm scatt. layer	CDCA	Cu(tmby)_2_ (TFSI)_1/2_ LiTFSI TBP	PEDOT	3.2	Osram Warm White 930	1000 (0.3031)	28 ± 2	[62]
**MS5** 329 (45,000) 463 (12,600)	**XY1b** 0.1 mM CDCA 2.5 mM CHCl_3_/EtOH 1:9 328 (34,000) 542 (36,400)	Cocktail **MS5**:**XY1b** 0.05:0.1mM CDCA 0.5mM CHCl_3_:EtOH 1:9 16 h	TiO_2_ 4 μm (transp.) 4 μm scatt. layer	CDCA	Cu(tmby)_2_ (TFSI)_1/2_ LiTFSI TBP	PEDOT	2.8	Osram Warm White 930	200	32.4	[15]
									500	32.4	
									1000	34.5	
**L1** 0.5 mM MeCN 404 (25,000)	**XY1** 0.01 mM CDCA 1mM CHCl_3_:EtOH 3:7 552 (56,500)	Cocktail **L1**:**XY1** 2.5:1 16 h	TiO_2_ 4μm (transp) 4 μm scatt. layer	CDCA	Cu(tmby)_2_ (TFSI)_1/2_ LiTFSI TBP	PEDOT	0.23	Osram Warm White 930	200	31.4	[63]
									500	32.7	
									1000 (0.3031)	34.0	
							3.2		1000 (0.3031)	33.2	
							8		1000 (0.3031)	30.6	
**D35** 0.1 mM MeCN:*t* BuOH 1:1 445 (70,100)	**XY1** 0.01 mM CDCA 1mM CHCl_3_:EtOH 3:7 552 (56,500)	Cocktail **D35**:**XY1** 4:1 16 h					-		1000 (0.3031)	29.5	[61]
**Y123** 0.2mM MeCN:*t*BuOH 1:1 530 (48,000)	**XY1b** 0.2 mM CDCA 5mM THF:EtOH 1:4 328 (34,000) 542 (36,400)	Cocktail **Y123**:**XY1b** 1:1 16 h					-		1000 (0.3031)	30.1	[64]
**SL9** 0.06mM in CHCl_3_:EtOH 2:8 330 (54,700) 557 (64,600) THF	**SL10** 0.1mM in MeCN:*t*BuOH 1:1 333 (41,900) 413 (33,000)	Cocktail: **SL9**:**SL10** 2:3 (0.06mM: 0.09mM) in CHCl_3_:EtOH 2:8 12 h	TiO_2_ 8 μm (transp.) 4 μm scatt. layer	BPHA pre-treat ment anti reflecting film	Cu(tmby)_2_ (TFSI)_1/2_ NaTFSI CEMI	PEDOT	2.8	T8 (4000K) LED tube	1479 (0.4310) 997 (0.2900) 859 (0.2520) 583 (0.1701) 201 (0.5898)	30.1 29.4 28.9 30.1 29.6	[65]
				BPHA pre-treat ment				T8 (3000 K) LED tube	936 (0.2758) 494 (0.1470)	29.5 30.2	
								T8 (6500 K) LED tube	949 (0.2869) 949 (0.2869)	28.4 29.1	
				none				T8 (4000K) LED tube	1479 (0.4310)	27.2	
								T8 (4000K) LED tube	1479 (0.4310)	26.3	

^(a)^ Light illuminance is reported in Lux; when available, the light power is reported in brackets. ^(b)^ When known, the color temperature is reported in parentheses.

## Data Availability

No new data were created.
